# Associations between obesity parameters and the risk of incident atrial fibrillation and ischaemic stroke in the different age groups

**DOI:** 10.3389/fcvm.2022.906844

**Published:** 2022-08-01

**Authors:** Hyo-Jeong Ahn, So-Ryoung Lee, Eue-Keun Choi, Kyung-Do Han, Tae-Min Rhee, Soonil Kwon, Sunwha Kim, Seil Oh, Gregory Y. H. Lip

**Affiliations:** ^1^Department of Internal Medicine, Seoul National University Hospital, Seoul, South Korea; ^2^Department of Internal Medicine, Seoul National University College of Medicine, Seoul, South Korea; ^3^Department of Statistics and Actuarial Science, Soongsil University, Seoul, South Korea; ^4^Presbyterian Medical Center, Jeonju, South Korea; ^5^Liverpool Centre for Cardiovascular Science, Liverpool Chest and Heart Hospital, University of Liverpool, Liverpool, United Kingdom; ^6^Department of Clinical Medicine, Aalborg University, Aalborg, Denmark

**Keywords:** body mass index, waist circumference, atrial fibrillation, ischaemic stroke, obesity

## Abstract

**Objective:**

Obesity and aging are important predisposing factors to atrial fibrillation (AF) and ischaemic stroke (IS). However, limited data comprehensively evaluated the relationships between obesity measurements and AF and IS in different ages.

**Methods:**

A total of 9,432,332 adults from the Korean National Health Insurance Service Database were included. The study population was categorized into the six age subgroups by an increase every decade from the twenties. We evaluated AF and IS risk according to body mass index (BMI) and waist circumference (WC) in the different age groups.

**Results:**

During a mean follow-up of 8.2 ± 1.0 years, BMI-AF presented a J-shaped association across ages. The highest hazard ratio (HR) of the BMI ≥ 30 kg/m^2^ group was observed in subjects aged 30–39 years [HR 1.80, 95% CI 1.63–1.98, *p* < 0.001]. Underweight adults over 60 years also presented an increased AF risk. Incident IS risk increased in those with BMI over the normal range in early and midlife, but the association became obscured in adults aged > 60 years. Among the BMI ≥ 30 kg/m^2^ groups, subjects aged 20–29 years presented the highest risk of IS [HR 3.00, 95% CI (2.34–3.84), *p* < 0.001]. Overall, WC-AF and WC-IS showed positive linear correlations, but the WC-IS association was weak in subjects aged ≥ 40 years.

**Conclusion:**

The higher risks of AF and IS according to an increment of BMI and WC were most apparent among the young ages. The association between obesity measurements and IS was not significantly above the midlife. Weight management in the young and integrated risk factor management in the elderly are warranted.

## Introduction

Obesity is a chronic disease and a global epidemic that threatens public health (1). It is associated with an increased risk of cardiovascular disease and reduced life expectancy (2, 3). Being underweight, as a part of malnutrition, is also related to adverse health consequences, namely, an increased risk of atrial fibrillation (AF), ischaemic stroke (IS), myocardial infarction (MI), and mortality (4–6). The degree of obesity can be evaluated in two representative ways: body mass index (BMI) and waist circumference (WC), indicating total body fat mass and visceral adiposity, respectively.

AF and IS are two representative debilitating cardiovascular diseases that share common attributable metabolic risk factors such as obesity. AF is the most common sustained cardiac arrhythmia of which the prevalence in adults is 2–4% (7, 8). The lifetime risk of AF has been recently estimated to be 1 in 3 in populations with European ancestry at an index age of 55 years owing to aging and increasing risk factor burden (9). AF is associated with an increased risk of stroke, heart failure, MI, dementia, and death, thus posing a significant burden on societal health and healthcare costs (10, 11). Stroke is another important condition that affects more than 15 million people each year and is the second leading cause of death worldwide (12, 13). Stroke is the most important cause of physical disability and dependency in adult life (14). The majority of strokes are attributed to modifiable metabolic or behavioral risk factors and related physiological changes such as high systolic blood pressure (15, 16). For both the AF and IS, aging is one of the major predisposing factors; the incidence of AF and IS increases with aging (8, 17).

Many studies have reported an association between various obesity measurements and the risk of AF and stroke. However, the associations stratified according to age, especially in Asia, where the rise in mean BMI of children and adolescents has accelerated since 2000, are unknown (4). This study aimed to evaluate the risk of AF and IS in the different age groups according to two obesity measurements, namely, BMI and WC, encompassing from underweight to overweight.

## Methods

### Data source and study population

We defined a nationwide population-based cohort from the National Health Information Database (NHID) (https://nhiss.nhis.or.kr/), integrating all the National Health Insurance Service Data, which covers the entire population of the Republic of Korea (hereafter, Korea). The NHID is only accessible online for authorized analysts at designated data centers with strict official payment release regulations. The database provides all the insurers' demographic information, income-based insurance contributions, medical usage information, including the International Classification of Diseases, Tenth Revision, Clinical Modification (ICD-10-CM), examination results, prescriptions, procedures, operation history, and inpatient and outpatient records. All the insured adults were eligible for a biennial general health examination. Health examination data included anthropometric measurements, laboratory variables, and responses to a self-reported questionnaire that asked about lifestyle behaviors such as smoking, alcohol intake, and physical activity (18–20). The Institutional Review Board of the Seoul National University Hospital (E-2108-077-1245) approved this study.

**Figure 1:**
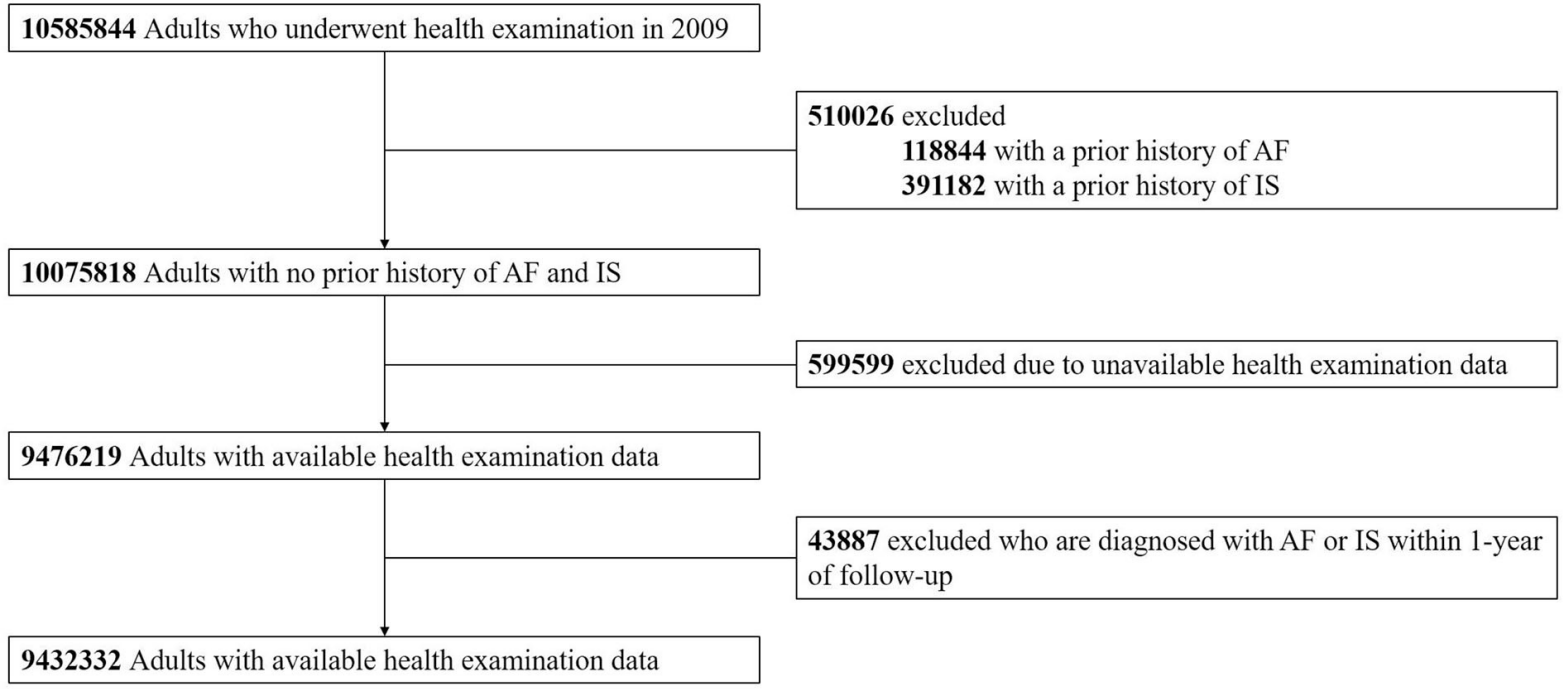
Study enrolment flow. AF, atrial fibrillation; IS, ischaemic stroke.

From 10,585,844 adults aged 20 years or older who underwent a biennially conducted regular health examination in 2009, individuals with a history of AF or IS and unavailable health examination data were excluded. Also, we excluded those diagnosed with AF or IS within 1 year of follow-up to reduce bias introduction from other predisposing and precipitating factors of the outcomes. Finally, we included 9,432,332 subjects in the analysis (Figure 1).

### Covariates

We retrieved individuals' baseline characteristics from a health examination report conducted in 2009. Comorbidities, such as hypertension, diabetes mellitus, dyslipidemia, MI, peripheral artery disease, chronic obstructive pulmonary disease, liver cirrhosis, chronic kidney disease, and cancer, were investigated according to the detailed definitions given in Supplementary Table 1 (20).

### Age subgroups and the categorization of obesity parameters

The study population was stratified by age into the following groups: 20–29, 30–39, 40–49, 50–59, 60–69, and 70 years or older. BMI was calculated as weight in kilograms divided by the square of height in meters (kg/m^2^). Regarding BMI, individuals were categorized into the 5 groups according to the classification suggested for Asians by the WHO Asia–Pacific Guidelines: underweight (BMI < 18.5 kg/m^2^), normal (≥18.5– < 23 kg/m^2^), overweight (≥23– < 25 kg/m^2^), obese class I (≥25– < 30 kg/m^2^), and obese class II (≥30 kg/m^2^) (21). WC was categorized into the 6 groups with distinctive points of 80, 85, 90, 95, and 100 cm for men and 75, 80, 85, 90, and 95 cm for women. Men with WC ≥ 90 cm and women with WC ≥ 85 cm were defined as having central obesity according to the Korean Society for the Study of Obesity (22).

### Follow-up and clinical outcomes

The incidence of AF and IS during follow-up was evaluated independently. Clinical outcomes were defined using the diagnostic codes (ICD-10-CM), incorporating inpatient and outpatient records: ICD-10-CM codes (I48.0–48.4, I48.9 for AF, and I63, I64 for IS), admission and outpatient department utilization history, along with additional brain imaging test records for IS. Detailed operational definitions of the outcomes are given in Supplementary Table 1 (20). The follow-up period was defined in two ways as the time between the baseline health examination date (index date) to the first occurrence of each clinical outcome (AF and IS), death, or the end of the study period (31 December 2018), whichever came first.

### Statistical analysis

Continuous variables are reported as mean ± SD and categorical variables are reported as numbers (%). The differences in baseline characteristics of each age group were evaluated using one-way ANOVA and the chi-square test. The crude incidence rates (IRs) of AF and IS were presented per 1,000 person-years (PY), dividing AF and IS cases by the total follow-up duration. We estimated the association between obesity measurements (BMI and WC) and primary outcomes (AF and IS) using the Cox proportional hazards regression models. According to BMI and WC categories, the risk of AF and IS was presented as hazard ratios (HRs) with 95% CIs. Model 1 is an unadjusted risk and model 2 was adjusted for age, sex, smoking status, alcohol intake, regular exercise, and low income. Model 3 was adjusted for comorbidities (hypertension, diabetes mellitus, dyslipidemia, MI, peripheral artery disease, chronic obstructive pulmonary disease, liver cirrhosis, chronic kidney disease, and cancer), and the Charlson Comorbidity Index, in addition to model 2. In each age group, the statistical significance (*p*-value) of the associations between obesity measurements (BMI and WC) and clinical outcomes (AF and IS) was assessed by calculating BMI and WC as continuous variables rather than a categorical variable that we predefined. Data collection and statistical analysis were performed using SAS version 9.4 (SAS Institute, Cary, NC, USA) from February 2021 to June 2021.

### Subgroup and sensitivity analyses

To examine potential effect modification, we performed subgroup analyses of the association between BMI/WC and the risk of AF/IS according to sex. In addition, sensitivity analysis was performed to verify the robustness of the results; the association was re-evaluated, excluding individuals diagnosed with AF or IS within the first 2 years of follow-up to minimize reverse causality.

## Results

The baseline characteristics of 9,432,332 adults stratified by age and BMI are shown in Table 1. The mean age was 46.4 ± 13.7 years and 5,172,446 (54.8%) were men. Mean BMI and WC were 23.7 ± 3.5 kg/m^2^ and 80.1 ± 9.5 cm, respectively.

**Table 1:** Baseline characteristics of the study population.

	**Total**	**Age 20-29 years**	**Age 30-39 years**	**Age 40-49 years**	**Age 50-59 years**	**Age 60-69 years**	**Age ≥70 years**	***P*** **-value**
***n***	9,432,332	1,184,942	1,878,710	2,576,067	2,008,736	1,195,123	5,88,754	
**Age, years**	46.4 ± 13.7	26.3 ± 2.3	34.5 ± 2.9	44.2 ± 3.0	53.8 ± 2.8	63.8 ± 2.9	74.2 ± 4.2	<0.001
**Sex**								<0.001
Male	5,172,446 (54.8)	602,746 (50.9)	1,365,919 (72.7)	1,370,213 (53.2)	992,012 (49.4)	580,369 (48.6)	261,187 (44.4)	
Female	4,259,886 (45.2)	582,196 (49.1)	512,791 (27.3)	1,205,854 (46.8)	1,016,724 (50.6)	614,754 (51.4)	327,567 (55.6)	
**Comorbidities**								
DM	762,031 (8.1)	11,469 (1.0)	53,298 (2.8)	154,487 (6.0)	228,498 (11.4)	202,812 (17.0)	111,467 (18.9)	<0.001
HTN	2,266,543 (24.0)	55,379 (4.7)	192,672 (10.3)	449,376 (17.4)	643,586 (32.0)	576,550 (48.2)	348,980 (59.3)	<0.001
DL	1,615,900 (17.1)	47,632 (4.0)	183,555 (9.8)	365,630 (14.2)	503,279 (25.1)	353,711 (29.6)	162,093 (27.5)	<0.001
CKD	605,657 (6.4)	48,678 (4.1)	72,986 (3.9)	125,561 (4.9)	110,376 (5.5)	138,838 (11.6)	109,218 (18.6)	<0.001
MI	20,697 (0.2)	275 (0.0)	999 (0.1)	3,306 (0.1)	5,960 (0.3)	6,026 (0.5)	4,131 (0.7)	<0.001
PAD	370,910 (3.9)	4,439 (0.4)	12,650 (0.7)	57,105 (2.2)	112,493 (5.6)	111,358 (9.3)	72,865 (12.4)	<0.001
COPD	325,539 (3.5)	19,097 (1.6)	36,781 (2.0)	63,282 (2.5)	77,030 (3.8)	74,789 (6.3)	54,560 (9.3)	<0.001
Cancer	112150 (1.2)	1564 (0.1)	5672 (0.3)	22763 (0.9)	34098 (1.7)	30078 (2.5)	17975 (3.1)	<0.001
LC	18287 (0.2)	229 (0.0)	1117 (0.1)	4213 (0.2)	6444 (0.3)	4684 (0.4)	1600 (0.3)	<0.001
**Lifestyle behaviors**								
Smoking								<0.001
Non	558,2105 (59.2)	725,012 (61.2)	834,805 (44.4)	1,504,459 (58.4)	1,267,584 (63.1)	815,887 (68.3)	434,358 (73.8)	
Ex	1,339,339 (14.2)	88,947 (7.5)	268,631 (14.3)	386,161 (15.0)	323,966 (16.1)	189,462 (15.9)	82,172 (14.0)	
Current	2,510,888 (26.6)	370,983 (31.3)	775,274 (41.3)	685,447 (26.6)	417,186 (20.8)	189774 (15.9)	72,224 (12.3)	
Drinking								<0.001
Non	4,784,812 (50.7)	434,733 (36.7)	674,233 (35.9)	1,240,758 (48.2)	1,172,354 (58.4)	812,581 (68.0)	450,153 (76.5)	
Mild	3,883,923 (41.2)	646,068 (54.5)	1,020,616 (54.3)	1,108,476 (43.0)	685,767 (34.1)	311,546 (26.1)	111,450 (18.9)	
Heavy	763,597 (8.1)	104,141 (8.8)	183,861 (9.8)	226,833 (8.8)	150,615 (7.5)	70,996 (5.9)	27,151 (4.6)	
Regular exericse	1,683,685 (17.9)	145,850 (12.3)	262,716 (14.0)	466,157 (18.1)	426500 (21.2)	279,473 (23.4)	102,989 (17.5)	<0.001
**Medications**								
Statin	786,478 (8.34)	4,103 (0.35)	30,745 (1.64)	129,635 (5.03)	270,070 (13.44)	238,678 (19.97)	113,247 (19.24)	<0.001
Aspirin	698,514 (7.41)	6,405 (0.54)	19,354 (1.03)	90,167 (3.5)	212,757 (10.59)	230,427 (19.28)	139,404 (23.68)	<0.001
Warfarin	3,952 (0.04)	86 (0.01)	354 (0.02)	740 (0.03)	977 (0.05)	1,016 (0.09)	779 (0.13)	<0.001
**CCI**	0.6 ± 1.1	0.2 ± 0.6	0.3 ± 0.7	0.5 ± 1.0	0.8 ± 1.2	1.1 ± 1.4	1.2 ± 1.5	<0.001
**Low income**	1,457,883 (15.5)	200,451 (16.9)	202,735 (10.8)	387,058 (15.0)	344,643 (17.2)	240,204 (20.1)	82,792 (14.1)	<0.001

*Data are presented as means ± standard deviations or number (percentages)*.

*Percentages may not total 100 because of rounding*.

*BMI, body mass index; DM, diabetes mellitus; HTN, hypertension; DL, dyslipidemia; CKD, chronic kidney disease; MI, myocardial infarction; PAD, peripheral artery disease; COPD, chronic obstructive pulmonary disease; LC, liver cirrhosis; CCI, Charlson Comorbidity Index*.

The outlines of the study population focusing on BMI—distribution of BMI, the proportion of sexes, lifestyle behaviors, comorbidities, and low-income contribution at each BMI according to age are given in Supplementary Figures 1–14. Of the total population, 3.8% were underweight, 39.5% were normal, and 56.7% were overweight or obese (24.5 % were overweight, 28.7% were obese class I, and 3.5% were obese class II). The proportion of overweight or obese individuals increased with age up to 60–69 years (Supplementary Figure 1). Males were predominant in overweight or obese individuals in their twenties and thirties; however, females accounted for the majority of these groups in the elderly (Supplementary Figure 1B). Regarding lifestyle behaviors, current smokers and heavy drinkers were the most prevalent in their thirties and forties. The gradual increase in the proportion of current smokers and heavy drinkers, with the increment of BMI at a young age, reversed after the forties and fifties. The proportion of regular exercisers was relatively lower in the young age group (~12–18% in the twenties to forties) than in the population group aged over 50 years (~17–23% in those in their fifties or older) (Supplementary Figures 2–4).

With respect to metabolic diseases and cardiovascular risk factors, such as diabetes mellitus, hypertension, dyslipidemia, MI, and peripheral artery disease, the prevalence increased with age. The tendency of a gradual increment in the proportion of the disease with an increase in BMI was maintained over the ages. In contrast, the proportions of diseases such as chronic obstructive pulmonary disease, liver cirrhosis, and cancer were higher in patients with lower BMI across the ages. The BMI distribution and the proportion of comorbidities at each BMI according to age are shown in Supplementary Figures 5–13. Low-income individuals with BMI over 23 kg/m^2^ increased with age, with 64.6% of the low-income population in their sixties being overweight or obese (Supplementary Figure 14).

### BMI, WC, and the risk of AF

**Figure 2:**
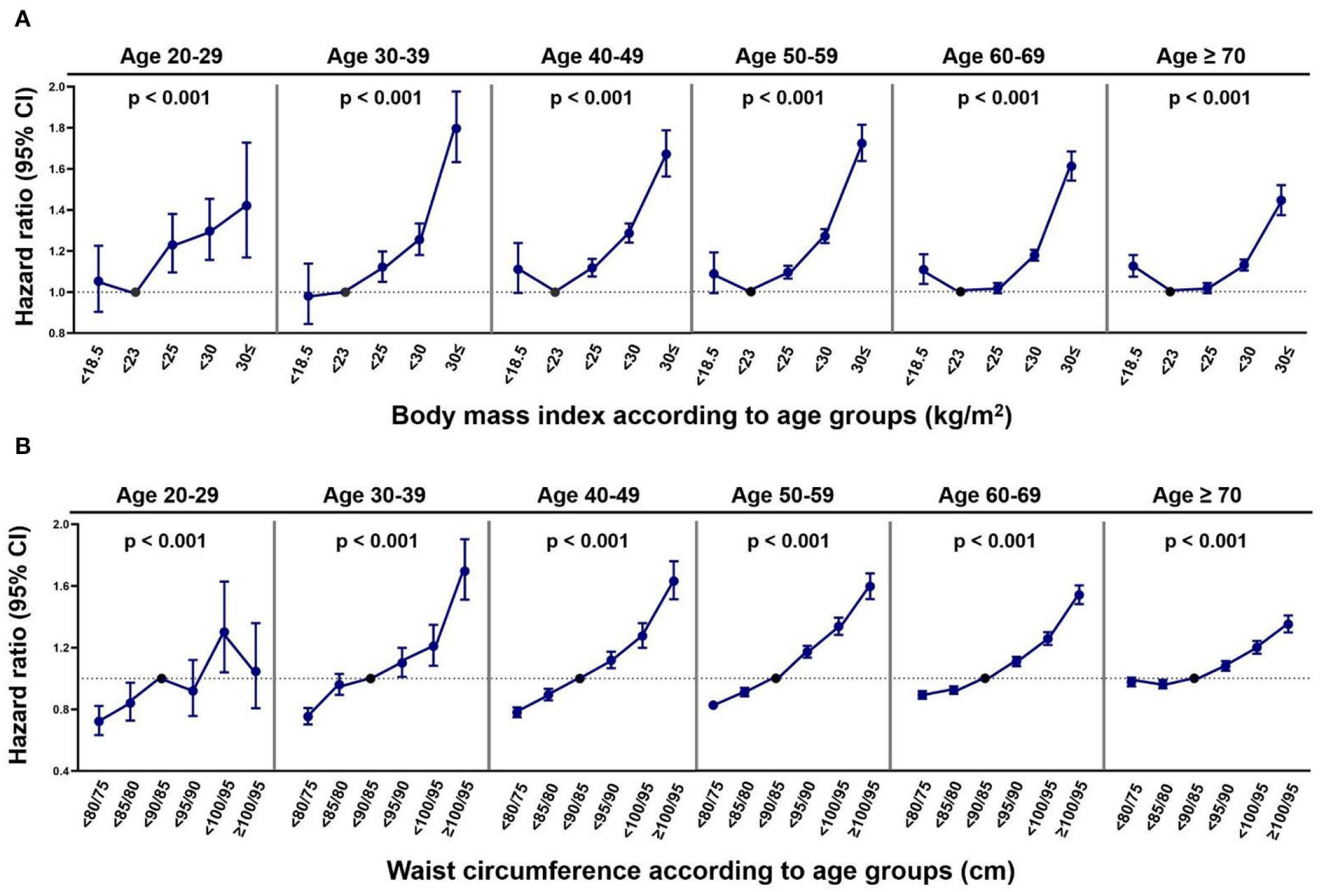
The risk of atrial fibrillation according to obesity parameters in the different age groups. **(A)** BMI. **(B)** WC. CI, confidence interval. *P*-values indicate the statistical significance of the association between obesity parameters (body mass index, waist circumference) and the risk of atrial fibrillation in each age group by calculating obesity parameters as continuous variables and adjusting covariates noted in model 3.

During the mean follow-up period of 8.2 ± 1.0 years, 155,558 subjects were diagnosed with new-onset AF (IR 2.02 per 1,000 PY). The event numbers, crude IRs, and HRs with 95% CIs of AF according to BMI and WC are given in Supplementary Tables 2, 3, respectively. The associations between BMI, WC, and the risk of AF in the different age groups adjusted by model 3 are shown in Figure 2.

Across all the age groups, individuals with BMI over the normal range were linearly associated with a higher risk of incident AF compared with those with normal BMI. The HR of obese class II (BMI ≥ 30 kg/m^2^) for the risk of AF compared with normal BMI was highest in subjects aged 30–39 years (HR 1.80, 95% CI 1.63–1.98) and attenuated in the younger and older age groups [HR (95% CI) 1.42 (1.17–1.73) in groups aged 20–29 years, 1.67 (1.56–1.79) in groups aged 40–49 years, 1.72 (1.64–1.81) in groups aged 50–59 years, 1.61 (1.54–1.68) in groups aged 60–69 years, and 1.45 (1.37–1.52) in groups aged 70 years or older].

The HRs of the underweight for the risk of incident AF were generally higher than normal BMI across the different age groups, but were only statistically significant in those over 60 years old [HR (95% CI) 1.11 (1.04–1.18) in those aged 60–69 years and 1.12 (1.07–1.18) in those aged 70 years or older].

Regarding WC and the risk of AF, a gradual increase in AF risk with an increase in WC was observed in all the age groups. The increased risk of incident AF in the highest WC group (≥100/95 cm) compared with the normal range of WC was most prominent in subjects aged 30–39 years (HR 1.70, 95% CI 1.51–1.90) and decreased with increasing age [HR (95% CI) 1.63 (1.51–1.76) in those aged 40–49 years, 1.60 (1.52–1.68) in those aged 50–59 years, 1.54 (1.48–1.60) in those aged 60–69 years, and 1.35 (1.30–1.41) in those aged 70 years or older]. In subjects aged 20–29 years, the association between high WC and the risk of AF was only significant in those with WC 95/90– < 100/95 cm. The low WC groups (WC < 80/75 cm or 80/75– < 85/80 cm) were associated with lower risks of AF across all the age groups, but this association tended to be markedly attenuated in the older age groups.

Subgroup analysis by sex is given in Supplementary Tables 4, 5. When adjusted by model 3, the associations between BMI and the risk of AF according to age were not modified by sex from the twenties to the fifties. The BMI-AF association at an age older than 60 years was modified by sex (*P*-for-interaction 0.01 and 0.02 for the sixties and age over 70 years, respectively). In contrast, the association between WC and the risk of AF according to age exhibited an interaction with sex at young ages, namely, 20–39 years (*P*-for-interaction was 0.02 and 0.03 for the twenties and the thirties, respectively). Supplementary Figures 15, 16 (adjusted HRs and CIs by model 3) summarize the associations differentiated by sex.

### BMI, WC, and the risk of IS

**Figure 3:**
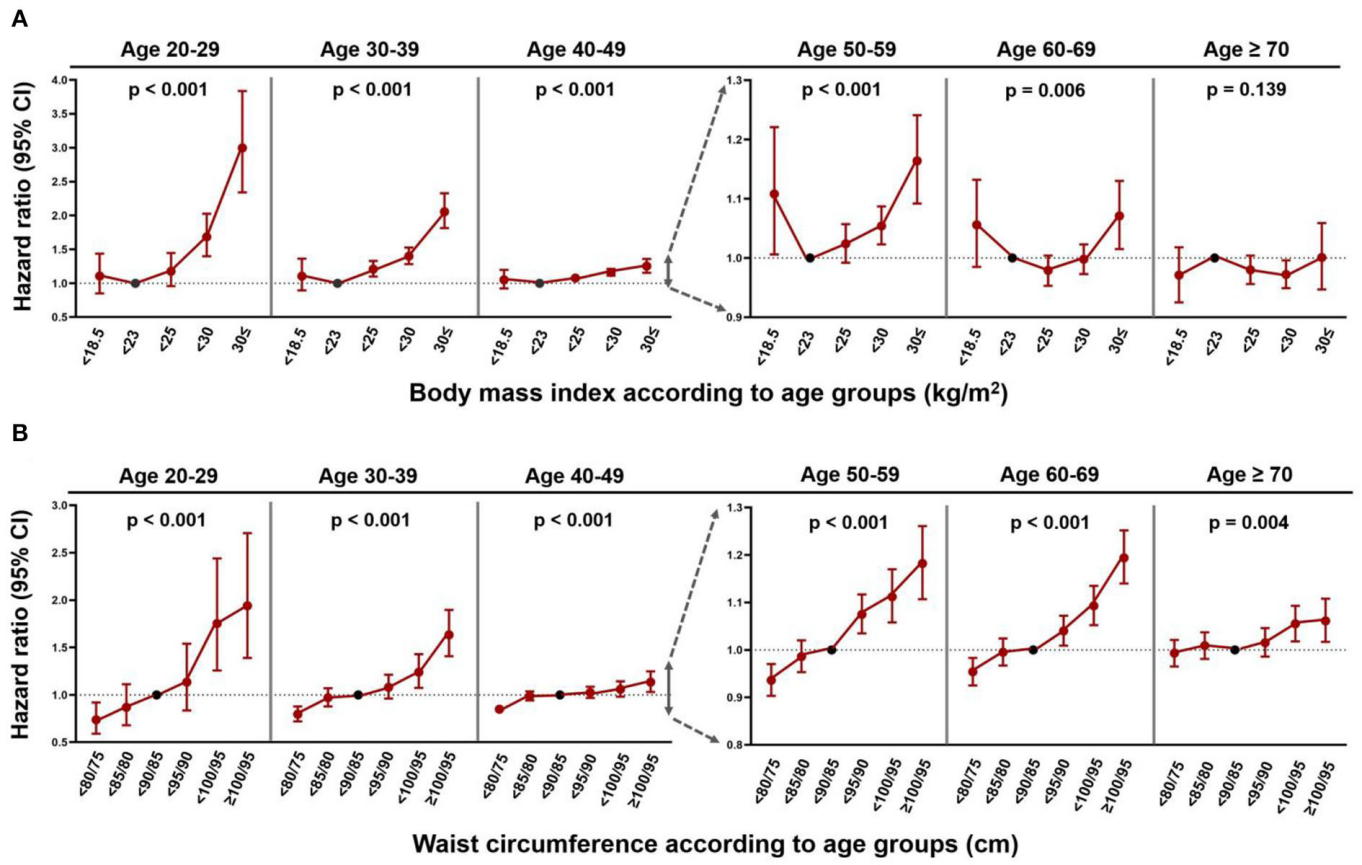
The risk of ischaemic stroke according to obesity parameters in the different age groups. **(A)** BMI. **(B)** WC. CI, confidence interval. *P*-values indicate the statistical significance of the association between obesity parameters (body mass index, waist circumference) and the risk of ischaemic stroke in each age group by calculating obesity parameters as continuous variables and adjusting covariates noted in model 3.

During a mean of 8.2 ± 1.0 years of follow-up, 128,501 subjects had IS. The crude IR was 1.66 per 1,000 PY. The event numbers, crude IRs, and HRs of IS according to BMI and WC in the different age groups are given in Supplementary Tables 6, 7, respectively. The associations between BMI, WC, and the risk of IS in the different age groups adjusted by model 3 are shown in Figure 3.

In patients aged between 20 and 59 years, individuals with a BMI over the normal range were positively associated with a higher risk of IS than those with a normal BMI. The highest HR for the risk of IS in obese class II (BMI ≥ 30 kg/m^2^) patients was observed in those aged 20–29 years (HR 3.00, 95% CI 2.34–3.84) and progressively decreased after ≥ 30 years [HR (95% CI) 2.06 (1.82–2.33) in those aged 30–39 years, 1.25 (1.16–1.36) in those aged 40–49 years, 1.16 (1.09–1.24) in those aged 50–59 years, 1.07 (1.02–1.13) in those aged 60–69 years, and 1.00 (0.95–1.06) in those aged 70 years or older]. The positive association between BMI and the risk of stroke was not significant in those aged > 60 years. Overall, IS risk was indeterminate for underweight individuals.

For WC and the risk of IS, a linear dose-response relationship was observed, but the association weakened in those aged > 40 years. The IS risk of individuals with WC ≥ 100/95 cm was highest in those aged 20–29 years (HR 1.94, 95% CI 1.39–2.71) and gradually reduced after ≥ 30 years of age [HR (95% CI) 1.63 (1.41–1.90) in those aged 30–39 years, 1.13 (1.03–1.25) in those aged 40–49 years, 1.18 (1.11–1.26) in those aged 50–59 years, 1.19 (1.14–1.25) in those aged 60–69 years, and 1.06 (1.02–1.11) in those aged 70 years or older]. Across the ages, the IS risk was lower in the lowest WC group (WC < 80/75 cm) compared with that in the normal WC group, but the association was not significant in those aged ≥ 70 years.

Subgroup analysis by sex is given in Supplementary Tables 8, 9 and is shown in Supplementary Figures 17, 18 (adjusted HRs and CIs by model 3). The risk of IS according to BMI showed an interaction with sex for the age group of 30–69 years (*P-for*-interactions were all <0.05). While males exhibited no significant relationship between BMI and IS from the age of 40 years, females showed a consistent positive correlation between BMI and IS risk up to the sixties. The association between WC and IS risk demonstrated interaction with sex between the age of 40 and 69 years. The linear correlation between WC and IS risk was more consistent in women than in men.

### Sensitivity analysis

The associations between obesity measurements (BMI and WC) and the risk of AF and IS, after excluding individuals diagnosed with AF and IS within the first 2 years of follow-up, are given in Supplementary Figures 19, 20. The overall J-shaped relationship of BMI-AF and BMI-IS (while attenuated among those over 60 years old), together with the positive linear correlations of WC-AF and WC-IS, remained unchanged.

## Discussion

Our nationwide cohort study is the first study to present the association between a complete set of BMI and WC categories and the risk of two representative cardiovascular diseases, AF and IS, in all the age groups of the adult population. Our major findings are as follows: (1) AF and IS showed different relationships with BMI among the age groups. The risk of AF had a J-shaped relationship with BMI and the increased risk of AF in underweight individuals was significant in those aged over 60 years; (2) a J-shaped relationship between BMI and the risk of IS was also observed, but this became attenuated and was not significant in individuals over 60 years; (3) WC and the risk of AF and IS were linearly correlated; (4) although there was statistical insignificance in the several age groups, being underweight, was associated with an increased risk of AF and IS in the general population; and (5) in both the AF and IS, the increased risk according to obesity measurements (BMI and WC) was most prominent at young ages.

We included an unprecedented amount of nationwide population-based data showing different demographic characteristics and the distribution of comorbidities in each age group. Data on demographics, comorbidities, and lifestyle behaviors were collected from nearly 9.5 million people. As given in Supplementary Figures 1–14, each age group demonstrated different characteristics. The proportion of sex and lifestyle behaviors in each BMI category showed a shift in trend as a reference to the middle-aged group. As expected, the prevalence of comorbidities in each BMI group increased with age. From these extensive data, age itself is clearly a health barometer that incorporates all the risk factors of cardiovascular disease. Indeed, individuals of different age strata would harbor distinctive risks of AF and IS according to predefined variables, such as BMI and WC.

Previous studies concluded that the relationship between BMI and the risk of AF is non-linear and best characterized as J-shaped (23–25). Higher BMI is well known to be associated with an increased risk of AF and being underweight has been identified as an independent risk factor for AF (6, 23). We have confirmed and extended prior findings, emphasizing different pathological BMI ranges according to age. A higher BMI confers an elevated risk of AF most prominently in the age 30–39 group, while being underweight is significantly linked to incident AF only in people over 60 years. Indeed, being underweight might have disparate biological meanings between the young and the old, as it could be a surrogate for frailty, sarcopenia, and malnutrition, all of which could act as a substrate for AF (5, 26, 27).

Overall, an increase in the risk of IS was observed as BMI increased and this association is in agreement with previous studies (28, 29). However, the J-shaped associations between BMI and IS were not uniform across the age, as it was only observed in young- and middle-aged adults and was attenuated in subjects over 60 years. Regarding underweight, the estimates of HRs were high, although they were only statistically significant in subjects aged 50–59 years. One previous study demonstrated a higher risk of stroke along with the severity of being underweight (5) and we could suggest that there might be a specific age range demonstrating excess IS risk associated with being underweight. Subgroup analysis revealed a more evident relationship between BMI and the risk of IS in women than in men; males showed a profound increase in IS risk with an increase in BMI up to subjects aged 30–39 years, whereas females exhibited a higher risk of IS consistently up to 60–69 years of age. Interestingly, age and sex are two critically important biological variables that influence the risk of stroke mediated by age-related sex hormones and endothelial responses (13). The non-significant relationship between BMI and IS risk among the older age groups suggests that other risk factors might play a major role in the development of IS in these patients. Different pathophysiologic mechanisms may lead to IS according to the age of onset. Indeed, above-average childhood BMI is positively associated with early-onset adult IS, but not with late-onset IS, inferring more dominating risk factors other than BMI contribute to IS later in life (30). Given that stroke is a debilitating disease and the leading cause of disability worldwide, (15, 31) maintaining a healthy weight is especially important in the young, while integrated management of risk factors should be considered in older individuals (32). Indeed, guidelines promote an integrated care approach for the management of AF, given the improved outcomes with such an approach (33).

In contrast to BMI, which exhibited a heterogeneous relationship with AF and IS according to age, WC presented a relatively uniform relationship with AF and IS across all the ages. The associations were both linear, showing no paradoxical increase in risk among patients with lower than normal WC. The degree of abdominal adiposity is well known to be linearly associated with AF (23) and WC and related ratios (such as waist-to-hip ratio) are reported to be more strongly associated with IS than BMI (34, 35). Thus, although BMI and WC are two indicators representing the degree of obesity, WC might be a better surrogate measure representing an unhealthy metabolic status and may add useful information to BMI to predict the risk of AF and IS (36, 37). Based on our results, we suggest that WC (an estimate of visceral fat) may predict future risk of cardiovascular disease better than BMI. In contrast, BMI could provide additional information on risk discrimination among underweight individuals, especially in the older age groups.

Obesity is increasing globally (1, 38) and is an important modifiable risk factor for AF and IS. Left atrial enlargement, of which BMI is a potent determinant, is an essential precursor of AF (39). It leads to AF *via* influence on myocardial structure and autonomic dysfunction, (40) endothelial dysfunction, and inflammatory and thrombogenic states, thereby affecting stroke risk (29, 31). As overweight, obesity, and the accompanying increased risk of AF and IS are preventable, efforts in population-based management of weight should be warranted with special attention to the young. Underweight should not be overlooked in the elderly because it could be a surrogate of malnutrition and lower lean body mass, which contribute to the development of cardiovascular disease.

### Limitations

Our study has several limitations. First, the event of AF may not represent the true incidence of AF. Since AF was ascertained using ICD-10-CM diagnostic codes and hospital utilization records rather than a 12-lead ECG, it might be underestimated, especially in the elderly. Second, the different risk associations of IS with the obesity parameters across the age might be affected by the preventive medication use since patients in the older age group are more likely to use oral anticoagulation or antiplatelet agents as per stroke risk prevention guidelines. Third, the evaluated associations have limited external generalisability to the other ethnic groups since the study population is Asian. Fourth, the results should be cautiously interpreted in the general population since those who could not undergo regular health examinations were not included. Fifth, there might be unadjusted confounders such as other comorbidities not included in the adjusted models. Lastly, long-term exposure and longitudinal variations of change in BMI and WC during the follow-up period might introduce bias in the associations.

## Conclusion

In these population-based data, we found a J-shaped association between BMI-AF and BMI-IS. We also noted positive linear correlations between WC-AF and WC-IS. The increased risk of AF and IS was most apparent in the young; hence, weight management in early life should receive due attention. Regarding IS, the association was not significant in the elderly, implicating that several factors other than obesity contribute to IS in this age group, thereby requiring integrated or holistic risk factor management.

## Data Availability Statement

The original contributions presented in the study are included in the article/Supplementary material, further inquiries can be directed to the corresponding author.

## Ethics Statement

The Institutional Review Board at the Seoul National University Hospital (E 2108-077-1245) authorized this study. Written informed consent was waived since anonymous and deidentified information was used for analysis.

## Author contributions

HJA, SRL, and EKC: conception and design. HJA, SRL, and KDH: acquisition of data. HJA, SRL, KDH, and EKC: analysis and interpretation of data. HJA: writing the manuscript. EKC and SO: administrative and technical or material supports. EKC: study guarantor. All authors contributed to the article and approved the submitted version.

## Funding

This study was supported by the Korea Medical Device Development Fund grant funded by the Korea Government (the Ministry of Science and ICT, the Ministry of Trade, Industry and Energy, the Ministry of Health and Welfare, Republic of Korea, and the Ministry of Food and Drug Safety) (Project Number: 202013B14) and by the Korea National Research Foundation funded by the Ministry of Education, Science and Technology (Grant 2020R1F1A106740).

## Conflict of interest

Authors EKC: Research grants or speaking fees from Bayer, BMS/Pfizer, Biosense Webster, Chong Kun Dang, Daiichi Sankyo, Dreamtech Co., Ltd., Medtronic, Samjin Pharmaceutical, Sanofi-Aventis, Seers Technology, and Skylabs. GL: Consultant and speaker for BMS/Pfizer, Boehringer Ingelheim, and Daiichi Sankyo. The remaining authors declare that the research was conducted in the absence of any commercial or financial relationships that could be construed as a potential conflict of interest.

## Publisher's note

All claims expressed in this article are solely those of the authors and do not necessarily represent those of their affiliated organizations, or those of the publisher, the editors and the reviewers. Any product that may be evaluated in this article, or claim that may be made by its manufacturer, is not guaranteed or endorsed by the publisher.
