# Supplementary material for: Associations between obesity parameters and the risk of incident atrial fibrillation and ischaemic stroke in the different age groups
Source: Front Cardiovasc Med. 2022 Aug 1;9:906844. doi: 10.3389/fcvm.2022.906844 (PMC9376314; doi:10.3389/fcvm.2022.906844)
Supplement: Supplementary file 1 [file Data_Sheet_1.docx]

**Associations between obesity parameters and the risk of incident atrial fibrillation and ischaemic stroke in different age groups: a nationwide population-based study**

Hyo-Jeong Ahn, MD^1*^; So-Ryoung Lee, MD, PhD^1*^; Eue-Keun Choi, MD, PhD^1,2^; Kyung-Do Han, PhD^3^;Tae-Min Rhee, MD^1^; Soonil Kwon, MD^1^; Sunwha Kim, MD^4^; Seil Oh, MD, PhD^1,2^; Gregory Y. H. Lip, MD, PhD ^2,5,6^

^1^ Department of Internal Medicine, Seoul National University Hospital, Seoul, Republic of Korea

^2^ Department of Internal Medicine, Seoul National University College of Medicine, Seoul, Republic of Korea

^3^ Department of Statistics and Actuarial Science, Soongsil University, Seoul, Republic of Korea

^4^ Presbyterian Medical Center, Jeonju, South Korea

^5^ Liverpool Centre for Cardiovascular Science, University of Liverpool and Liverpool Chest & Heart Hospital, Liverpool, United Kingdom

^6^ Department of Clinical Medicine, Aalborg University, Aalborg, Denmark

* These authors have contributed equally to this work

**Correspondence to:**

Eue-Keun Choi, MD, PhD

Department of Internal Medicine, Seoul National University Hospital

101 Daehak-ro, Jongno-gu, Seoul, 03080, Republic of Korea

Phone +82-2-2072-0688/Fax +82-2-762-9662

E-mail: choiek17@snu.ac.kr

**Supplementary Tables and Figures**

Supplementary Table 1. Definition of covariates and outcomes.

Supplementary Table 2. The risk of atrial fibrillation according to body mass index among different age groups.

Supplementary Table 3. The risk of atrial fibrillation according to waist circumference among different age groups.

Supplementary Table 4. The association between body mass index and the risk of atrial fibrillation according to age differentiated by sex.

Supplementary Table 5. The association between waist circumference and the risk of atrial fibrillation according to age differentiated by sex.

Supplementary Table 6. The risk of ischaemic stroke according to body mass index among different age groups.

Supplementary Table 7. The risk of ischaemic stroke according to waist circumference among different age groups.

Supplementary Table 8. The association between body mass index and the risk of ischaemic stroke according to age differentiated by sex.

Supplementary Table 9. The association between waist circumference and the risk of ischaemic stroke according to age differentiated by sex.

Supplementary Figure 1. A) The distribution of BMI and according to age, B) The distribution of sex at each BMI according to age.

Supplementary Figure 2. A) The BMI distribution of current smokers, B) proportion of current smokers at each BMI according to age.

Supplementary Figure 3. A) The BMI distribution of heavy drinkers, B) proportion of heavy drinkers at each BMI according to age.

Supplementary Figure 4. A) The BMI distribution of regular exercisers, B) proportion of regular exercisers at each BMI according to age.

Supplementary Figure 5. A) The BMI distribution of diabetes mellitus, B) proportion of diabetes mellitus at each BMI according to age.

Supplementary Figure 6. A) The BMI distribution of hypertension, B) proportion of hypertension at each BMI according to age.

Supplementary Figure 7. A) The BMI distribution of dyslipidemia, B) proportion of dyslipidemia at each BMI according to age.

Supplementary Figure 8. A) The BMI distribution of CKD, B) proportion of CKD at each BMI according to age.

Supplementary Figure 9. A) The BMI distribution of MI, B) proportion of MI at each BMI according to age.

Supplementary Figure 10. A) The BMI distribution of PAD, B) proportion of PAD at each BMI according to age.

Supplementary Figure 11. A) The BMI distribution of COPD, B) proportion of COPD at each BMI according to age.

Supplementary Figure 12. A) The BMI distribution of cancer, B) proportion of cancer at each BMI according to age.

Supplementary Figure 13. A) The BMI distribution of LC, B) proportion of LC at each BMI according to age.

Supplementary Figure 14. A) The BMI distribution of low income, B) proportion of low income at each BMI according to age.

Supplementary Figure 15. The association between body mass index and the risk of atrial fibrillation according to age differentiated by sex.

Supplementary Figure 16. The association between waist circumference and the risk of atrial fibrillation according to age differentiated by sex.

Supplementary Figure 17. The association between body mass index and the risk of ischaemic stroke according to age differentiated by sex.

Supplementary Figure 18. The association between waist circumference and the risk of ischaemic stroke according to age differentiated by sex.

Supplementary Figure 19. The association between obesity measurements (BMI, WC) and the risk of atrial fibrillation excluding individuals diagnosed with atrial fibrillation within the first two years of follow-up.

Supplementary Figure 20. The association between obesity measurements (BMI, WC) and the risk of ischaemic stroke excluding individuals diagnosed with ischaemic stroke within the first two years of follow-up.

**Tables**

Supplementary Table 1. Definition of covariates and outcomes

| **Diagnosis** | **ICD-10-CM code and medication** | | | **Number of diagnosis** | **Diagnosis test or treatment** | | **Combination*** |
| --- | --- | --- | --- | --- | --- | --- | --- |
| **Outcome** |  | | |  |  | |  |
| Atrial fibrillation | I48.0-48.4, I48.9 | | | Admission ≥ 1 or outpatient department ≥ 2 | N/A | | 1+2 |
| Ischaemic stroke | I63, I64 | | | Admission ≥ 1 or outpatient department ≥ 1 | Brain imaging (CT or MRI) ≥1 or  Concomitant imaging studies of the brain or related death | | 1+2+3 |
| **Comorbidities based on the health examination** | | | | |  | |  |
| **Comorbidities** |  | | |  |  | |  |
| Hypertension | I10-I13, I15; and minimum 1 prescription of anti-hypertensive drug (thiazide, loop diuretics, aldosterone antagonist, alpha-/beta-blocker, calcium-channel blocker, angiotensin-converting enzyme inhibitor, or angiotensin II receptor blocker) | | | Admission ≥ 1 or outpatient department ≥ 2 | Systolic/diastolic blood pressure ≥ 140/90 mmHg | | 1+2 or 3 |
| Diabetes mellitus | E11-E14; and minimum 1 prescription of anti-diabetic drugs (sulfonylureas, metformin, meglitinides, thiazolidinediones, dipeptidyl peptidase-4 inhibitors, α-glucosidase inhibitors, or insulin) | | | Admission ≥ 1 or outpatient department ≥ 2 | Fasting glucose level ≥ 126 mg/dL | | 1+2 or 3 |
| Dyslipidemia | E78 | | | Admission ≥ 1 or outpatient department ≥ 1 | Total cholesterol ≥ 240 mg/dL | | 1+2 or 3 |
| MI | I21, I22 | | | Admission ≥ 1 or outpatient department ≥ 1 | N/A | | 1+2 |
| PAD | I70, I73 | | | Admission ≥ 1 or outpatient department ≥ 1 | N/A | | 1+2 |
| COPD | J41-44 | | | Admission ≥ 1 | N/A | | 1+2 |
| CKD | N/A | | | N/A | eGFR<60ml/min/1.73m^2^ | | 3 |
| LC | K74, K702, K703, K717, K761 | | | Admission ≥ 1 or outpatient department ≥ 2 | N/A | | 1+2 |
| Cancer | C00-97 and RID code (V193) | | | Admission ≥ 1 or outpatient department ≥ 1 | N/A | | 1+2 |
| **Definitions of life style behavior** | | | | | | | |
| **Alcohol consumption** |  | | |  |  | |  |
| Mild drinker | Alcohol consumption > 0g to < 30g per day | | | |  | |  |
| Heavy drinker | Alcohol consumption ≥ 30g per day | | | |  | |  |
| **Smoking** |  | | |  |  | |  |
| Ex-smoker | Ex-smoker at the health examination | | | | | | |
| Current smoker | Current smoker at the health examination | | | | | | |
| **Regular exercise** | Performing > 30 min of moderate physical activity ≥ five times a week or > 20 min of vigorous physical activity ≥ three times a week | | | | | | |
| **Clinical score (Charlson comorbidity index)** | | | | | | | |
| **Category** | | **Weights** | **Disease** | | | **ICD-10-CM code** | |
| **Myocardial infarction** | | 1 | Acute myocardial infarction | | | I21 | |
|  |  |  | Subsequent myocardial infarction | | | I22 | |
| **Congestive heart failure** | | 1 | Heart Failure | | | I50 | |
| **Peripheral vascular disease** | | 1 | Atherosclerosis | | | I70 | |
|  |  |  | Other peripheral vascular disease | | | I73 | |
| **Cerebrovascular disease** | | 1 | Transient cerebral ischemic attacks and related syndromes | | | G45 | |
|  |  |  | Vascular syndromes of brain in cerebrovascular diseases | | | G46 | |
|  |  |  | Retinal vascular occlusion | | | H34 | |
|  |  |  | Cerebrovascular disease | | | I60-I69 | |
| **Dementia** | | 1 | Dementia in Alzheimer disease | | | F00 | |
|  |  |  | Vascular dementia | | | F01 | |
|  |  |  | Dementia in other disease classified elsewhere | | | F02 | |
|  |  |  | Unspecified dementia | | | F03 | |
| **Chronic pulmonary disease** | | 1 | Chronic lower respiratory diseases | | | J40-J47 | |
|  |  |  | Lung disease due to external agents | | | J60-J67 | |
| **Rheumatic disease**  **(connective tissue disorder)** | | 1 | Rheumatoid arthritis with rheumatoid factor | | | M05 | |
|  |  |  | Felty's syndrome | | | M05.0 | |
|  |  |  | Rheumatoid lung disease with rheumatoid arthritis | | | M05.1 | |
|  |  |  | Rheumatoid vasculitis with rheumatoid arthritis | | | M05.2 | |
|  |  |  | Rheumatoid heart disease with rheumatoid arthritis | | | M05.3 | |
|  |  |  | Rheumatoid myopathy with rheumatoid arthritis | | | M05.4 | |
|  |  |  | Rheumatoid polyneuropathy with rheumatoid arthritis | | | M05.5 | |
|  |  |  | Rheumatoid arthritis with involvement of other organs and systems | | | M05.6 | |
|  |  |  | Rheumatoid arthritis with rheumatoid factor without organ or systems involvement | | | M05.7 | |
|  |  |  | Other rheumatoid arthritis with rheumatoid factor | | | M05.8 | |
|  |  |  | Rheumatoid arthritis without rheumatoid factor | | | M05.9 | |
|  |  |  | Adult-onset Still's disease | | | M06.1 | |
|  |  |  | Rheumatoid bursitis | | | M06.2 | |
|  |  |  | Rheumatoid nodule | | | M06.3 | |
|  |  |  | Inflammatory polyarthropathy | | | M06.4 | |
|  |  |  | Other specified rheumatoid arthritis | | | M06.8 | |
|  |  |  | Rheumatoid arthritis, unspecified | | | M06.9 | |
|  |  |  | Giant cell arteritis with polymyalgia rheumatica | | | M31.5 | |
|  |  |  | Systemic lupus erythematosus (SLE) | | | M32 | |
|  |  |  | Drug-induced SLE | | | M32.0 | |
|  |  |  | SLE with organ or system involvement | | | M32.1 | |
|  |  |  | Other forms of SLE | | | M32.8 | |
|  |  |  | SLE, unspecified | | | M32.9 | |
|  |  |  | Dermatopolymyositis | | | M33 | |
|  |  |  | Juvenile dermatomyositis | | | M33.0 | |
|  |  |  | Other dermatomyositis | | | M33.1 | |
|  |  |  | Polymyositis | | | M33.2 | |
|  |  |  | Dermatopolymyositis, unspecified | | | M33.9 | |
|  |  |  | Systemic sclerosis [scleroderma] | | | M34 | |
|  |  |  | Progressive systemic sclerosis | | | M34.0 | |
|  |  |  | CR(E)ST syndrome | | | M34.1 | |
|  |  |  | Systemic sclerosis induced by drug and chemical | | | M34.2 | |
|  |  |  | Other forms of systemic sclerosis | | | M34.8 | |
|  |  |  | Systemic sclerosis, unspecified | | | M34.9 | |
|  |  |  | Other overlap syndromes | | | M35.1 | |
|  |  |  | Polymyalgia rheumatica | | | M35.3 | |
|  |  |  | Dermato(poly)myositis in neoplastic disease | | | M36.0 | |
| **Peptic ulcer disease** | | 1 | Gastric ulcer | | | K25 | |
|  |  |  | Duodenal ulcer | | | K26 | |
|  |  |  | Peptic ulcer, site unspecified | | | K27 | |
|  |  |  | Gastrojejunal ulcer | | | K28 | |
| **Mild liver disease** | | 1 | Chronic viral hepatitis | | | B18 | |
|  |  |  | Alcoholic fatty liver | | | K70.0- K70.3, K70.9 | |
|  |  |  | Alcoholic hepatitis | | |  | |
|  |  |  | Alcoholic fibrosis and sclerosis of liver | | |  | |
|  |  |  | Alcoholic cirrhosis of liver | | |  | |
|  |  |  | Alcoholic liver disease, unspecified | | |  | |
|  |  |  | Toxic liver disease with chronic persistent hepatitis | | | K71.3- K71.5, K71.7 | |
|  |  |  | Toxic liver disease with chronic lobular hepatitis | | |  | |
|  |  |  | Toxic liver disease with chronic active hepatitis | | |  | |
|  |  |  | Toxic liver disease with fibrosis and cirrhosis of liver | | |  | |
|  |  |  | Chronic hepatitis, not elsewhere classified | | | K73 | |
|  |  |  | Fibrosis and cirrhosis of liver | | | K74 | |
|  |  |  | Fatty (change of) liver, not elsewhere classified | | | K76.0-K76.4, K76.8, K76.9 | |
|  |  |  | Nonalcoholic fatty liver disease | | |  | |
|  |  |  | Central hemorrhagic necrosis of liver | | |  | |
|  |  |  | Infarction of liver | | |  | |
|  |  |  | Hepatic angiomatosis | | |  | |
|  |  |  | Other specified disease of liver | | |  | |
|  |  |  | Simple cyst of liver | | |  | |
|  |  |  | Focal nodular hyperplasia of liver | | |  | |
|  |  |  | Hepatoptosis | | |  | |
|  |  |  | Liver disease, unspecified | | |  | |
|  |  |  | Liver transplant status | | | Z94.4 | |
| **Diabetes without chronic**  **complication** | | 1 | with coma | | | E10.0, 10.1, 10.6, 10.8, 10.9 | |
|  |  |  | with ketoacidosis | | | E11.0, 11.1, 11.6, 11.8, 11.9 | |
|  |  |  | with other specified complications | | | E12.0, 12.1, 12.6, 12.8, 12.9 | |
|  |  |  | with unspecified complications | | | E13.0, 13.1, 13.6, 13.8, 13.9 | |
|  |  |  | without complications | | | E14.0, 14.1, 14.6, 14.8, 14.9 | |
| **Diabetes with chronic**  **complication** | | 2 | with renal complications | | | E10.2, 10.3, 10.4, 10.5, 10.7 | |
|  |  |  | with ophthalmic complications | | | E11.2, 11.3, 11.4, 11.5, 11.7 | |
|  |  |  | with neurologic complications | | | E12.2, 12.3, 12.4, 12.5, 12.7 | |
|  |  |  | with peripheral circulatory complications | | | E13.2, 13.3, 13.4, 13.5, 13.7 | |
|  |  |  | with multiple complications | | | E14.2, 14.3, 14.4, 14.5, 14.7 | |
| **Hemi/paraplegia** | | 2 | Tropical spastic paraplegia | | | G04.1 | |
|  |  |  | Hereditary spastic paraplegia | | | G11.4 | |
|  |  |  | Spastic quadriplegic cerebral palsy | | | G80.0 | |
|  |  |  | Spastic diplegic cerebral palsy | | | G80.1 | |
|  |  |  | Spastic hemiplegic cerebral palsy | | | G80.2 | |
|  |  |  | Flaccid hemiplegia | | | G81.0 | |
|  |  |  | Spastic hemiplegia | | | G81.1 | |
|  |  |  | Hemiplegia, unspecified | | | G81.9 | |
|  |  |  | Flaccid paraplegia | | | G82.0 | |
|  |  |  | Spastic paraplegia | | | G82.1 | |
|  |  |  | Paraplegia, unspecified | | | G82.2 | |
|  |  |  | Flaccid tetraplegia | | | G82.3 | |
|  |  |  | Spastic tetraplegia | | | G82.4 | |
|  |  |  | Tetraplegia, unspecified | | | G82.5 | |
|  |  |  | Diplegia of upper limbs | | | G83.0 | |
|  |  |  | Paralytic syndrome, unspecified | | | G83.9 | |
| **Renal disease** | | 2 | Hypertensive renal disease | | | I12 | |
|  |  |  | Hypertensive heart and renal disease with renal failure | | | I13.1 | |
|  |  |  | Chronic nephritic syndrome | | | N03 | |
|  |  |  | Unspecified nephritic syndrome | | | N05 | |
|  |  |  | Chronic kidney disease | | | N18 | |
|  |  |  | Unspecified kidney failure | | | N19 | |
|  |  |  | Disorders resulting from impaired renal tubular function | | | N25 | |
|  |  |  | Care involving dialysis | | | Z49 | |
|  |  |  | Transplanted organ and tissue status - kidney | | | Z94.0 | |
|  |  |  | Dependence on renal dialysis | | | Z99.2 | |
| **Cancer** | | 2 | Any tumor, malignant neoplasm | | | C00-76, C97 | |
|  |  |  | Any tumor, in situ neoplasm | | | D00-09 | |
|  |  |  | Any tumor, Benign neoplasm | | | D10-36 | |
|  |  |  | Any tumor, Neoplasm of unknown behavior | | | D37-48 | |
|  |  |  | Leukemia | | | C91-95 | |
|  |  |  | Lymphoma | | | C81-86 | |
| **Metastatic cancer** | | 3 | Metastatic solid tumor | | | C77-80 | |
| **Moderate to severe**  **liver disease** | | 3 | Esophageal varices | | | I85 | |
|  |  |  | Gastric varices | | | I86.4 | |
|  |  |  | Esophageal varices without bleeding in diseases classified elsewhere | | | I98.2 | |
|  |  |  | Alcoholic hepatic failure | | | K70.4 | |
|  |  |  | Toxic liver disease with hepatic necrosis | | | K71.1 | |
|  |  |  | Hepatic failure (acute/chronic) due to drugs | | |  | |
|  |  |  | Chronic hepatic failure | | | K72.1, K72.9 | |
|  |  |  | Hepatic failure, unspecified | | |  | |
|  |  |  | Hepatic veno-occlusive disease | | | K76.5-K76.7 | |
|  |  |  | Portal hypertension | | |  | |
|  |  |  | Hepatorenal syndrome | | |  | |
| **Human immunodeficiency**  **Virus (HIV)** | | 6 | HIV disease resulting in infectious and parasitic diseases | | | B20 | |
|  |  |  | HIV disease resulting in malignant neoplasm | | | B21 | |
|  |  |  | HIV disease resulting in other specified diseases | | | B22 | |
|  |  |  | HIV disease resulting in other conditions | | | B23 | |
| **Information of income** | | | | | | | |
| Low income | Income belongs to lower 20% among the entire Korean population and supported by the Medical Aid program | | | | | | |

Abbreviation: N/A, not applicable; DM, diabetes mellitus; HTN, hypertension; DL, dyslipidemia; CKD, chronic kidney disease; MI, myocardial infarction; PAD, peripheral artery disease; COPD, chronic obstructive pulmonary disease; LC, liver cirrhosis.

* Combination: 1= ICD-10-CM code and medication; 2 = Number of diagnosis; and 3 = Diagnostic tests or treatment

Supplementary Table 2. The risk of atrial fibrillation according to body mass index among different age groups.

| Age | BMI | Number | Events | IR  (1000 PY) | Model 1 HR (95% CI) | Model 2 HR (95% CI) | Model 3 HR (95% CI) |
| --- | --- | --- | --- | --- | --- | --- | --- |
| 20-29 | <18.5 | 131414 | 204 | 0.187 | 0.954 (0.821-1.109) | 1.051 (0.903-1.225) | 1.053 (0.904-1.226) |
|  | <23 | 629174 | 1023 | 0.196 | 1 (Ref.) | 1 (Ref.) | 1 (Ref.) |
|  | <25 | 193269 | 442 | 0.276 | 1.408 (1.260-1.574) | 1.251 (1.115-1.403) | 1.230 (1.096-1.380) |
|  | <30 | 191129 | 495 | 0.313 | 1.599 (1.436-1.780) | 1.379 (1.232-1.543) | 1.297 (1.157-1.454) |
|  | ≥30 | 39956 | 126 | 0.382 | 1.954 (1.624-2.351) | 1.725 (1.430-2.081) | 1.421 (1.169-1.728) |
|  | p-value |  |  |  | <0.001 | <0.001 | <0.001 |
| 30-39 | <18.5 | 81204 | 190 | 0.282 | 0.819 (0.706-0.950) | 0.980 (0.844-1.137) | 0.981 (0.845-1.139) |
|  | <23 | 765984 | 2188 | 0.345 | 1 (Ref.) | 1 (Ref.) | 1 (Ref.) |
|  | <25 | 421422 | 1585 | 0.454 | 1.318 (1.236-1.406) | 1.145 (1.072-1.222) | 1.122 (1.050-1.198) |
|  | <30 | 525545 | 2395 | 0.551 | 1.602 (1.511-1.697) | 1.340 (1.263-1.423) | 1.255 (1.181-1.334) |
|  | ≥30 | 84555 | 594 | 0.854 | 2.483 (2.268-2.718) | 2.160 (1.970-2.367) | 1.797 (1.633-1.977) |
|  | p-value |  |  |  | <0.001 | <0.001 | <0.001 |
| 40-49 | <18.5 | 58200 | 339 | 0.708 | 1.018 (0.913-1.136) | 1.094 (0.981-1.221) | 1.111 (0.996-1.239) |
|  | <23 | 1033176 | 5946 | 0.695 | 1 (Ref.) | 1 (Ref.) | 1 (Ref.) |
|  | <25 | 658174 | 4964 | 0.912 | 1.312 (1.263-1.362) | 1.158 (1.115-1.203) | 1.118 (1.076-1.162) |
|  | <30 | 743641 | 7161 | 1.167 | 1.680 (1.624-1.739) | 1.418 (1.369-1.468) | 1.287 (1.241-1.334) |
|  | ≥30 | 82876 | 1074 | 1.578 | 2.274 (2.131-2.426) | 2.087 (1.955-2.227) | 1.672 (1.563-1.788) |
|  | p-value |  |  |  | <0.001 | <0.001 | <0.001 |
| 50-59 | <18.5 | 32671 | 488 | 1.848 | 1.100 (1.005-1.205) | 1.058 (0.966-1.159) | 1.087 (0.993-1.191) |
|  | <23 | 692868 | 9600 | 1.684 | 1 (Ref.) | 1 (Ref.) | 1 (Ref.) |
|  | <25 | 562230 | 9366 | 2.022 | 1.201 (1.167-1.236) | 1.136 (1.104-1.169) | 1.094 (1.063-1.126) |
|  | <30 | 656746 | 13710 | 2.540 | 1.509 (1.470-1.549) | 1.390 (1.354-1.427) | 1.270 (1.237-1.305) |
|  | ≥30 | 64221 | 1802 | 3.428 | 2.039 (1.939-2.144) | 2.067 (1.965-2.173) | 1.722 (1.636-1.813) |
|  | p-value |  |  |  | <0.001 | <0.001 | <0.001 |
| 60-69 | <18.5 | 24448 | 958 | 5.127 | 1.150 (1.077-1.228) | 1.071 (1.003-1.144) | 1.107 (1.037-1.182) |
|  | <23 | 379612 | 13670 | 4.491 | 1 (Ref.) | 1 (Ref.) | 1 (Ref.) |
|  | <25 | 331487 | 12445 | 4.638 | 1.031(1.007-1.057) | 1.058 (1.032-1.084) | 1.017 (0.992-1.042) |
|  | <30 | 418028 | 18474 | 5.463 | 1.215 (1.188-1.242) | 1.277 (1.248-1.305) | 1.177 (1.151-1.204) |
|  | ≥30 | 41548 | 2427 | 7.272 | 1.619 (1.550-1.690) | 1.858 (1.779-1.941) | 1.611 (1.541-1.683) |
|  | p-value |  |  |  | <0.001 | <0.001 | <0.001 |
| 70- | <18.5 | 28494 | 2010 | 10.987 | 1.165 (1.112-1.220) | 1.089 (1.039-1.141) | 1.124 (1.073-1.178) |
|  | <23 | 222453 | 15503 | 9.598 | 1 (Ref.) | 1 (Ref.) | 1 (Ref.) |
|  | <25 | 146864 | 10701 | 9.661 | 1.001 (0.977-1.026) | 1.054 (1.028-1.080) | 1.017 (0.992-1.042) |
|  | <30 | 173364 | 13943 | 10.558 | 1.093 (1.068-1.118) | 1.206 (1.178-1.234) | 1.130 (1.103-1.157) |
|  | ≥30 | 17579 | 1735 | 13.022 | 1.350 (1.284-1.418) | 1.606 (1.528-1.689) | 1.445 (1.373-1.519) |
|  | p-value |  |  |  | <0.001 | <0.001 | <0.001 |
| P-for-interaction | |  |  |  | <0.001 | <0.001 | <0.001 |

Abbreviation: BMI, body mass index; IR, incidence rate; PY, person-years; HR, hazard ratio; CI, confidence interval.

Model 1 is an unadjusted risk.

Model 2 was adjusted for age, sex, smoking status (never smoker, ex-smoker, or current smoker), alcohol intake (non, mild, or heavy drinker, g/day), regular exercise (performing > 30 min of moderate physical activity ≥ five times a week or > 20 min of vigorous physical activity ≥ three times a week), and low-income level (income in lower 20% among the entire Korean population thus supported by the medical aid program).

Model 3 was adjusted for comorbidities (hypertension, diabetes mellitus, dyslipidemia, myocardial infarction, peripheral artery disease, chronic obstructive pulmonary disease, liver cirrhosis, chronic kidney disease, and cancer), and frailty index, Charlson Comorbidity Index (CCI), in addition to Model 2.

Supplementary Table 3. The risk of atrial fibrillation according to waist circumference among different age groups.

| Age | WC (M/F) | Number | Events | IR  (1000PY) | Model 1 HR (95% CI) | Model 2 HR (95% CI) | Model 3 HR (95% CI) |
| --- | --- | --- | --- | --- | --- | --- | --- |
| 20-29 | <80/75 | 759354 | 1217 | 0.1929 | 0.594 (0.524-0.673) | 0.691 (0.607-0.787) | 0.722 (0.633-0.822) |
|  | <85/80 | 203556 | 440 | 0.2604 | 0.803 (0.694-0.928) | 0.820 (0.709-0.949) | 0.841 (0.727-0.973) |
|  | <90/85 | 114921 | 309 | 0.3243 | 1 (Ref.) | 1 (Ref.) | 1 (Ref.) |
|  | <95/90 | 57784 | 149 | 0.3112 | 0.960 (0.789-1.167) | 0.953 (0.784-1.159) | 0.920 (0.757-1.12) |
|  | <100/95 | 27223 | 103 | 0.457 | 1.410 (1.129-1.763) | 1.409 (1.127-1.761) | 1.302 (1.040-1.629) |
|  | ≥100/95 | 22104 | 72 | 0.3941 | 1.217 (0.941-1.572) | 1.233 (0.954-1.594) | 1.047 (0.807-1.359) |
|  | p-value |  |  |  | <0.001 | <0.001 | <0.001 |
| 30-39 | <80/75 | 812481 | 2117 | 0.3143 | 0.599 (0.560-0.641) | 0.721 (0.672-0.773) | 0.754 (0.703-0.809) |
|  | <85/80 | 450044 | 1773 | 0.4756 | 0.907 (0.846-0.973) | 0.936 (0.872-1.004) | 0.960 (0.894-1.03) |
|  | <90/85 | 319693 | 1386 | 0.524 | 1 (Ref.) | 1 (Ref.) | 1 (Ref.) |
|  | <95/90 | 173521 | 866 | 0.604 | 1.153 (1.059-1.255) | 1.138 (1.046-1.239) | 1.101 (1.011-1.199) |
|  | <100/95 | 75640 | 425 | 0.6808 | 1.300 (1.166-1.450) | 1.299 (1.166-1.449) | 1.209 (1.083-1.348) |
|  | ≥100/95 | 47331 | 385 | 0.9887 | 1.892 (1.690-2.118) | 1.968 (1.757-2.203) | 1.696 (1.512-1.903) |
|  | p-value |  |  |  | <0.001 | <0.001 | <0.001 |
| 40-49 | <80/75 | 1009746 | 5371 | 0.6425 | 0.596 (0.573-0.62) | 0.728 (0.699-0.759) | 0.780 (0.749-0.813) |
|  | <85/80 | 657560 | 4838 | 0.8899 | 0.826 (0.793-0.86) | 0.864 (0.830-0.900) | 0.895 (0.859-0.933) |
|  | <90/85 | 496234 | 4415 | 1.0775 | 1 (Ref.) | 1 (Ref.) | 1 (Ref.) |
|  | <95/90 | 259688 | 2760 | 1.2895 | 1.197 (1.142-1.256) | 1.167 (1.113-1.224) | 1.119 (1.067-1.174) |
|  | <100/95 | 102164 | 1276 | 1.5177 | 1.410 (1.325-1.500) | 1.392 (1.308-1.481) | 1.276 (1.199-1.359) |
|  | ≥100/95 | 50675 | 824 | 1.9832 | 1.845 (1.713-1.988) | 1.920 (1.782-2.068) | 1.632 (1.514-1.760) |
|  | p-value |  |  |  | <0.001 | <0.001 | <0.001 |
| 50-59 | <80/75 | 572883 | 7129 | 1.5101 | 0.675 (0.654-0.697) | 0.773 (0.749-0.798) | 0.826 (0.800-0.853) |
|  | <85/80 | 527734 | 8307 | 1.9118 | 0.855 (0.830-0.881) | 0.882 (0.856-0.909) | 0.910 (0.883-0.938) |
|  | <90/85 | 470867 | 8659 | 2.2355 | 1 (Ref.) | 1 (Ref.) | 1 (Ref.) |
|  | <95/90 | 270789 | 6188 | 2.7853 | 1.246 (1.206-1.288) | 1.212 (1.173-1.252) | 1.172 (1.135-1.211) |
|  | <100/95 | 112482 | 2975 | 3.2295 | 1.446 (1.387-1.507) | 1.429 (1.371-1.49) | 1.337 (1.282-1.394) |
|  | ≥100/95 | 53981 | 1708 | 3.8813 | 1.739 (1.651-1.831) | 1.798 (1.707-1.894) | 1.597 (1.515-1.682) |
|  | p-value |  |  |  | <0.001 | <0.001 | <0.001 |
| 60-69 | <80/75 | 253884 | 8426 | 4.1371 | 0.849 (0.826-0.873) | 0.836 (0.813-0.859) | 0.892 (0.867-0.917) |
|  | <85/80 | 282834 | 10136 | 4.4389 | 0.910 (0.886-0.934) | 0.897 (0.874-0.921) | 0.923 (0.899,0.948) |
|  | <90/85 | 305944 | 12072 | 4.8793 | 1 (Ref.) | 1 (Ref.) | 1 (Ref.) |
|  | <95/90 | 204903 | 9236 | 5.5913 | 1.146 (1.116-1.178) | 1.139 (1.108-1.170) | 1.110 (1.080-1.140) |
|  | <100/95 | 97556 | 4982 | 6.3454 | 1.301 (1.259-1.345) | 1.323 (1.280-1.368) | 1.258 (1.217-1.300) |
|  | ≥100/95 | 50002 | 3122 | 7.8381 | 1.610 (1.547-1.674) | 1.678 (1.613-1.746) | 1.542 (1.482-1.604) |
|  | p-value |  |  |  | <0.001 | <0.001 | <0.001 |
| 70- | <80/75 | 136347 | 9258 | 9.5005 | 0.994 (0.967-1.023) | 0.924 (0.898-0.951) | 0.976 (0.948-1.004) |
|  | <85/80 | 125003 | 8651 | 9.3238 | 0.971 (0.943-0.999) | 0.939 (0.913-0.967) | 0.962 (0.935-0.990) |
|  | <90/85 | 139070 | 10058 | 9.6222 | 1 (Ref.) | 1 (Ref.) | 1 (Ref.) |
|  | <95/90 | 101377 | 8011 | 10.5142 | 1.093 (1.062-1.126) | 1.104 (1.072-1.137) | 1.080 (1.049-1.112) |
|  | <100/95 | 54712 | 4765 | 11.6039 | 1.207 (1.166-1.249) | 1.248 (1.205-1.291) | 1.201 (1.160-1.243) |
|  | ≥100/95 | 32245 | 3149 | 13.1519 | 1.371 (1.317-1.427) | 1.438 (1.381-1.497) | 1.352 (1.298-1.408) |
|  | p-value |  |  |  | <0.001 | <0.001 | <0.001 |
| p for interaction | |  |  |  | <0.001 | <0.001 | <0.001 |

Abbreviation: WC, waist circumference; M, male; F, female; IR, incidence rate; PY, person-years; HR, hazard ratio; CI, confidence interval.

Model 1 is an unadjusted risk.

Model 2 was adjusted for age, sex, smoking status (never smoker, ex-smoker, or current smoker), alcohol intake (non, mild, or heavy drinker, g/day), regular exercise (performing > 30 min of moderate physical activity ≥ five times a week or > 20 min of vigorous physical activity ≥ three times a week), and low-income level (income in lower 20% among the entire Korean population thus supported by the medical aid program).

Model 3 was adjusted for comorbidities (hypertension, diabetes mellitus, dyslipidemia, myocardial infarction, peripheral artery disease, chronic obstructive pulmonary disease, liver cirrhosis, chronic kidney disease, and cancer), and frailty index, Charlson Comorbidity Index (CCI), in addition to Model 2.

Supplementary Table 4. The association between body mass index and the risk of atrial fibrillation according to age differentiated by sex

| Age | Sex | BMI | Number | Event | IR  (1000PY) | Model 1 | p for  interaction | Model 3 | p for  interaction |
| --- | --- | --- | --- | --- | --- | --- | --- | --- | --- |
| 20-29 | Male | <18.5 | 22155 | 33 | 0.17949 | 0.773 (0.543-1.099) | 0.094 | 0.799 (0.562-1.137) | 0.165 |
|  |  | <23 | 261354 | 505 | 0.23247 | 1 (Ref.) |  | 1 (Ref.) |  |
|  |  | <25 | 139549 | 356 | 0.30724 | 1.322 (1.155-1.514) |  | 1.262 (1.101-1.447) |  |
|  |  | <30 | 150415 | 427 | 0.34257 | 1.476 (1.297-1.679) |  | 1.335 (1.170-1.524) |  |
|  |  | ≥30 | 29273 | 97 | 0.40127 | 1.732 (1.393-2.152) |  | 1.393 (1.110-1.747) |  |
|  |  | p-value |  |  |  | <0.001 |  | <0.001 |  |
|  | Female | <18.5 | 109259 | 171 | 0.18822 | 1.110 (0.934-1.320) |  | 1.111 (0.934-1.321) |  |
|  |  | <23 | 367820 | 518 | 0.16949 | 1 (Ref.) |  | 1 (Ref.) |  |
|  |  | <25 | 53720 | 86 | 0.19291 | 1.138 (0.906-1.430) |  | 1.123 (0.893-1.411) |  |
|  |  | <30 | 40714 | 68 | 0.20145 | 1.189 (0.923-1.531) |  | 1.125 (0.871-1.453) |  |
|  |  | ≥30 | 10683 | 29 | 0.32785 | 1.936 (1.332-2.814) |  | 1.546 (1.044-2.288) |  |
|  |  | p-value |  |  |  | 0.008 |  | 0.181 |  |
| 30-39 | Male | <18.5 | 28273 | 87 | 0.3717 | 0.899 (0.724-1.115) | 0.365 | 0.934 (0.753-1.160) | 0.548 |
|  |  | <23 | 453456 | 1556 | 0.41367 | 1 (Ref.) |  | 1 (Ref.) |  |
|  |  | <25 | 347683 | 1414 | 0.49071 | 1.187 (1.104-1.275) |  | 1.131 (1.052-1.216) |  |
|  |  | <30 | 464738 | 2237 | 0.58219 | 1.409 (1.321-1.503) |  | 1.269 (1.188-1.356) |  |
|  |  | ≥30 | 71769 | 551 | 0.93322 | 2.262 (2.052-2.492) |  | 1.855 (1.676-2.054) |  |
|  |  | p-value |  |  |  | <0.001 |  | <0.001 |  |
|  | Female | <18.5 | 52931 | 103 | 0.23455 | 0.961 (0.780-1.184) |  | 1.007 (0.817-1.241) |  |
|  |  | <23 | 312528 | 632 | 0.24399 | 1 (Ref.) |  | 1 (Ref.) |  |
|  |  | <25 | 73739 | 171 | 0.28029 | 1.149 (0.970-1.360) |  | 1.090 (0.920-1.292) |  |
|  |  | <30 | 60807 | 158 | 0.31437 | 1.289 (1.083-1.534) |  | 1.162 (0.972-1.388) |  |
|  |  | ≥30 | 12786 | 43 | 0.40734 | 1.670 (1.226-2.274) |  | 1.335 (0.968-1.841) |  |
|  |  | p-value |  |  |  | <0.001 |  | 0.249 |  |
| 40-49 | Male | <18.5 | 21116 | 163 | 0.94709 | 0.994 (0.849-1.163) | 0.493 | 1.032 (0.882-1.208) | 0.505 |
|  |  | <23 | 422685 | 3325 | 0.95443 | 1 (Ref.) |  | 1 (Ref.) |  |
|  |  | <25 | 383071 | 3528 | 1.11621 | 1.170 (1.115-1.226) |  | 1.112 (1.060-1.166) |  |
|  |  | <30 | 496090 | 5579 | 1.36624 | 1.433 (1.372-1.496) |  | 1.284 (1.229-1.342) |  |
|  |  | ≥30 | 47251 | 772 | 1.99778 | 2.098 (1.940-2.269) |  | 1.721 (1.588-1.866) |  |
|  |  | p-value |  |  |  | <0.001 |  | <0.001 |  |
|  | Female | <18.5 | 37084 | 176 | 0.57326 | 1.109 (0.952-1.292) |  | 1.180 (1.013-1.375) |  |
|  |  | <23 | 610491 | 2621 | 0.51714 | 1 (Ref.) |  | 1 (Ref.) |  |
|  |  | <25 | 275103 | 1436 | 0.62907 | 1.216 (1.141-1.297) |  | 1.130 (1.059-1.206) |  |
|  |  | <30 | 247551 | 1582 | 0.77106 | 1.491 (1.401-1.588) |  | 1.289 (1.209-1.375) |  |
|  |  | ≥30 | 35625 | 302 | 1.0259 | 1.985 (1.763-2.237) |  | 1.539 (1.361-1.740) |  |
|  |  | p-value |  |  |  | <0.001 |  | <0.001 |  |
| 50-59 | Male | <18.5 | 15769 | 312 | 2.50832 | 1.068 (0.953-1.196) | 0.001 | 1.079 (0.963-1.209) | 0.193 |
|  |  | <23 | 304282 | 5832 | 2.35856 | 1 (Ref.) |  | 1 (Ref.) |  |
|  |  | <25 | 288030 | 6231 | 2.64865 | 1.122 (1.083-1.163) |  | 1.085 (1.046-1.124) |  |
|  |  | <30 | 358778 | 9318 | 3.18577 | 1.351 (1.307-1.396) |  | 1.241 (1.200-1.283) |  |
|  |  | ≥30 | 25153 | 960 | 4.7323 | 2.010 (1.877-2.152) |  | 1.694 (1.580-1.816) |  |
|  |  | p-value |  |  |  | <0.001 |  | <0.001 |  |
|  | Female | <18.5 | 16902 | 176 | 1.2603 | 1.081 (0.929-1.257) |  | 1.101 (0.946-1.280) |  |
|  |  | <23 | 388586 | 3768 | 1.16681 | 1 (Ref.) |  | 1 (Ref.) |  |
|  |  | <25 | 274200 | 3135 | 1.37522 | 1.178 (1.124-1.236) |  | 1.101 (1.049-1.154) |  |
|  |  | <30 | 297968 | 4392 | 1.77555 | 1.522 (1.457-1.589) |  | 1.311 (1.254-1.371) |  |
|  |  | ≥30 | 39068 | 842 | 2.60862 | 2.238 (2.077-2.411) |  | 1.733 (1.605-1.871) |  |
|  |  | p-value |  |  |  | <0.001 |  | <0.001 |  |
| 60-69 | Male | <18.5 | 14401 | 647 | 6.11429 | 1.090 (1.007-1.181) | <.0001 | 1.079 (0.996-1.169) | 0.008 |
|  |  | <23 | 198003 | 8772 | 5.65773 | 1 (Ref.) |  | 1 (Ref.) |  |
|  |  | <25 | 165615 | 7589 | 5.76484 | 1.017 (0.986-1.049) |  | 1.000 (0.969-1.032) |  |
|  |  | <30 | 190746 | 10232 | 6.75504 | 1.192 (1.158-1.226) |  | 1.141 (1.108-1.175) |  |
|  |  | ≥30 | 11604 | 882 | 9.74951 | 1.725 (1.609-1.848) |  | 1.578 (1.471-1.693) |  |
|  |  | p-value |  |  |  | <0.001 |  | <0.001 |  |
|  | Female | <18.5 | 10047 | 311 | 3.8373 | 1.174 (1.047-1.317) |  | 1.155 (1.030-1.296) |  |
|  |  | <23 | 181609 | 4898 | 3.27956 | 1 (Ref.) |  | 1 (Ref.) |  |
|  |  | <25 | 165872 | 4856 | 3.55301 | 1.083 (1.041-1.127) |  | 1.045 (1.004-1.087) |  |
|  |  | <30 | 227282 | 8242 | 4.41437 | 1.346 (1.299-1.395) |  | 1.229 (1.185-1.274) |  |
|  |  | ≥30 | 29944 | 1545 | 6.35027 | 1.941 (1.833-2.055) |  | 1.644 (1.551-1.742) |  |
|  |  | p-value |  |  |  | <0.001 |  | <0.001 |  |
| 70- | Male | <18.5 | 14630 | 1156 | 13.1832 | 1.194 (1.123-1.270) | 0.347 | 1.177 (1.106-1.252) | 0.024 |
|  |  | <23 | 110723 | 8652 | 11.2652 | 1 (Ref.) |  | 1 (Ref.) |  |
|  |  | <25 | 67372 | 5544 | 11.3253 | 0.999 (0.966-1.033) |  | 0.997 (0.964-1.032) |  |
|  |  | <30 | 64898 | 6061 | 12.7708 | 1.126 (1.089-1.163) |  | 1.110 (1.073-1.148) |  |
|  |  | ≥30 | 3564 | 419 | 16.3763 | 1.449 (1.314-1.599) |  | 1.393 (1.262-1.538) |  |
|  |  | p-value |  |  |  | <0.001 |  | <.0001 |  |
|  | Female | <18.5 | 13864 | 854 | 8.9659 | 1.126 (1.048-1.209) |  | 1.055 (0.982-1.133) |  |
|  |  | <23 | 111730 | 6851 | 8.0867 | 1 (Ref.) |  | 1 (Ref.) |  |
|  |  | <25 | 79492 | 5157 | 8.343 | 1.028 (0.991-1.066) |  | 1.040 (1.003-1.079) |  |
|  |  | <30 | 108466 | 7882 | 9.3171 | 1.148 (1.111-1.185) |  | 1.150 (1.113-1.189) |  |
|  |  | ≥30 | 14015 | 1316 | 12.2248 | 1.510 (1.423-1.601) |  | 1.472 (1.386-1.563) |  |
|  |  | p-value |  |  |  | <0.001 |  | <0.001 |  |

Abbreviation: WC, waist circumference; IR, incidence rate; PY, person-years; HR, hazard ratio; CI, confidence interval.

Model 1 is an unadjusted risk.

Model 3 was adjusted for age, sex, smoking status (never smoker, ex-smoker, or current smoker), alcohol intake (non, mild, or heavy drinker, g/day), regular exercise (performing > 30 min of moderate physical activity ≥ five times a week or > 20 min of vigorous physical activity ≥ three times a week), comorbidities (hypertension, diabetes mellitus, dyslipidemia, myocardial infarction, peripheral artery disease, chronic obstructive pulmonary disease, liver cirrhosis, chronic kidney disease, and cancer), low-income level (income in lower 20% among the entire Korean population thus supported by the medical aid program), and frailty index, Charlson Comorbidity Index (CCI).

Supplementary Table 5. The association between waist circumference and the risk of atrial fibrillation according to age differentiated by sex.

| Age | Sex | WC | Number | Event | IR  (1000PY) | Model 1 | p for  interaction | Model 3 | p for  interaction |
| --- | --- | --- | --- | --- | --- | --- | --- | --- | --- |
| 20-29 | Male | <80/75 | 287581 | 536 | 0.224 | 0.607 (0.524-0.702) | 0.010 | 0.658 (0.567-0.764) | 0.019 |
|  |  | <85/80 | 145984 | 339 | 0.280 | 0.757 (0.645-0.888) |  | 0.782 (0.666-0.918) |  |
|  |  | <90/85 | 87530 | 268 | 0.369 | 1 (Ref.) |  | 1 (Ref.) |  |
|  |  | <95/90 | 44969 | 132 | 0.354 | 0.960 (0.779-1.183) |  | 0.928 (0.753-1.144) |  |
|  |  | <100/95 | 20669 | 91 | 0.532 | 1.442 (1.137-1.829) |  | 1.340 (1.055-1.702) |  |
|  |  | ≥100/95 | 16013 | 52 | 0.393 | 1.067 (0.793-1.436) |  | 0.924 (0.683-1.249) |  |
|  |  | p-value |  |  |  | <0.001 |  | <0.001 |  |
|  | Female | <80/75 | 471773 | 681 | 0.174 | 0.962 (0.702-1.318) |  | 1.007 (0.734-1.382) |  |
|  |  | <85/80 | 57572 | 101 | 0.211 | 1.171 (0.815-1.684) |  | 1.200 (0.835-1.726) |  |
|  |  | <90/85 | 27391 | 41 | 0.181 | 1 (Ref.) |  | 1 (Ref.) |  |
|  |  | <95/90 | 12815 | 17 | 0.160 | 0.886 (0.503-1.559) |  | 0.853 (0.485-1.502) |  |
|  |  | <100/95 | 6554 | 12 | 0.221 | 1.224 (0.643-2.329) |  | 1.116 (0.585-2.128) |  |
|  |  | ≥100/95 | 6091 | 20 | 0.396 | 2.201 (1.290-3.755) |  | 1.768 (1.025-3.048) |  |
|  |  | p-value |  |  |  | 0.005 |  | 0.122 |  |
| 30-39 | Male | <80/75 | 457870 | 1405 | 0.370 | 0.661 (0.613-0.713) | 0.004 | 0.723 (0.670-0.781) | 0.025 |
|  |  | <85/80 | 372054 | 1586 | 0.514 | 0.919 (0.854-0.989) |  | 0.956 (0.888-1.029) |  |
|  |  | <90/85 | 277241 | 1284 | 0.560 | 1 (Ref.) |  | 1 (Ref.) |  |
|  |  | <95/90 | 153723 | 811 | 0.638 | 1.141 (1.045-1.246) |  | 1.099 (1.006-1.200) |  |
|  |  | <100/95 | 65821 | 404 | 0.744 | 1.330 (1.189-1.488) |  | 1.239 (1.107-1.386) |  |
|  |  | ≥100/95 | 39210 | 355 | 1.101 | 1.973 (1.754-2.219) |  | 1.738 (1.542-1.959) |  |
|  |  | p-value |  |  |  | <0.001 |  | <0.001 |  |
|  | Female | <80/75 | 354611 | 712 | 0.242 | 0.833 (0.677-1.025) |  | 0.905 (0.734-1.115) |  |
|  |  | <85/80 | 77990 | 187 | 0.290 | 0.997 (0.783-1.269) |  | 1.026 (0.806-1.306) |  |
|  |  | <90/85 | 42452 | 102 | 0.291 | 1 (Ref.) |  | 1 (Ref.) |  |
|  |  | <95/90 | 19798 | 55 | 0.336 | 1.156 (0.833-1.605) |  | 1.115 (0.803-1.549) |  |
|  |  | <100/95 | 9819 | 21 | 0.259 | 0.891 (0.557-1.425) |  | 0.831 (0.519-1.330) |  |
|  |  | ≥100/95 | 8121 | 30 | 0.447 | 1.540 (1.025-2.313) |  | 1.352 (0.894-2.045) |  |
|  |  | p-value |  |  |  | 0.002 |  | 0.152 |  |
| 40-49 | Male | <80/75 | 373046 | 2673 | 0.869 | 0.691 (0.657-0.727) | 0.422 | 0.769 (0.731-0.81) | 0.502 |
|  |  | <85/80 | 389608 | 3455 | 1.075 | 0.855 (0.815-0.896) |  | 0.896 (0.854-0.939) |  |
|  |  | <90/85 | 326229 | 3379 | 1.257 | 1 (Ref.) |  | 1 (Ref.) |  |
|  |  | <95/90 | 181005 | 2223 | 1.494 | 1.189 (1.127-1.254) |  | 1.135 (1.076-1.197) |  |
|  |  | <100/95 | 69248 | 1016 | 1.788 | 1.424 (1.327-1.527) |  | 1.306 (1.217-1.401) |  |
|  |  | ≥100/95 | 31077 | 621 | 2.448 | 1.953 (1.793-2.127) |  | 1.698 (1.557-1.852) |  |
|  |  | p-value |  |  |  | <0.001 |  | <0.001 |  |
|  | Female | <80/75 | 636700 | 2698 | 0.511 | 0.695 (0.647-0.746) |  | 0.789 (0.734-0.849) |  |
|  |  | <85/80 | 267952 | 1383 | 0.622 | 0.846 (0.781-0.917) |  | 0.891 (0.822-0.966) |  |
|  |  | <90/85 | 170005 | 1036 | 0.735 | 1 (Ref.) |  | 1 (Ref.) |  |
|  |  | <95/90 | 78683 | 537 | 0.823 | 1.120 (1.009-1.243) |  | 1.064 (0.959-1.181) |  |
|  |  | <100/95 | 32916 | 260 | 0.954 | 1.299 (1.133-1.488) |  | 1.177 (1.027-1.349) |  |
|  |  | ≥100/95 | 19598 | 203 | 1.254 | 1.709 (1.470-1.986) |  | 1.444 (1.240-1.682) |  |
|  |  | p-value |  |  |  | <0.001 |  | <0.001 |  |
| 50-59 | Male | <80/75 | 222646 | 3877 | 2.142 | 0.759 (0.728-0.790) | 0.137 | 0.824 (0.791-0.858) | 0.517 |
|  |  | <85/80 | 273519 | 5534 | 2.479 | 0.878 (0.846-0.910) |  | 0.912 (0.879-0.946) |  |
|  |  | <90/85 | 257398 | 5930 | 2.824 | 1 (Ref.) |  | 1 (Ref.) |  |
|  |  | <95/90 | 154978 | 4304 | 3.414 | 1.209 (1.163-1.258) |  | 1.158 (1.114-1.205) |  |
|  |  | <100/95 | 59342 | 1983 | 4.124 | 1.462 (1.389-1.538) |  | 1.346 (1.279-1.417) |  |
|  |  | ≥100/95 | 24129 | 1025 | 5.288 | 1.877 (1.757-2.006) |  | 1.639 (1.533-1.752) |  |
|  |  | p-value |  |  |  | <0.001 |  | <0.001 |  |
|  | Female | <80/75 | 350237 | 3252 | 1.117 | 0.726 (0.690-0.764) |  | 0.837 (0.795-0.881) |  |
|  |  | <85/80 | 254215 | 2773 | 1.312 | 0.853 (0.809-0.899) |  | 0.908 (0.861-0.958) |  |
|  |  | <90/85 | 213469 | 2729 | 1.539 | 1 (Ref.) |  | 1 (Ref.) |  |
|  |  | <95/90 | 115811 | 1884 | 1.961 | 1.274 (1.202-1.351) |  | 1.206 (1.137-1.279) |  |
|  |  | <100/95 | 53140 | 992 | 2.253 | 1.465 (1.362-1.575) |  | 1.314 (1.221-1.413) |  |
|  |  | ≥100/95 | 29852 | 683 | 2.774 | 1.805 (1.660-1.963) |  | 1.525 (1.401-1.660) |  |
|  |  | p-value |  |  |  | <0.001 |  | <0.001 |  |
| 60-69 | Male | <80/75 | 130444 | 5249 | 5.140 | 0.851 (0.821-0.881) | 0.192 | 0.890 (0.859-0.923) | 0.564 |
|  |  | <85/80 | 147261 | 6450 | 5.533 | 0.914 (0.884-0.945) |  | 0.937 (0.906-0.969) |  |
|  |  | <90/85 | 146963 | 7060 | 6.054 | 1 (Ref.) |  | 1 (Ref.) |  |
|  |  | <95/90 | 97868 | 5360 | 6.935 | 1.146 (1.106-1.187) |  | 1.113 (1.074-1.154) |  |
|  |  | <100/95 | 40398 | 2587 | 8.152 | 1.348 (1.289-1.41) |  | 1.274 (1.218-1.333) |  |
|  |  | ≥100/95 | 17435 | 1416 | 10.523 | 1.744 (1.648-1.847) |  | 1.585 (1.497-1.679) |  |
|  |  | p-value |  |  |  | <0.001 |  | <0.001 |  |
|  | Female | <80/75 | 123440 | 3177 | 3.128 | 0.817 (0.781-0.854) |  | 0.896 (0.856-0.937) |  |
|  |  | <85/80 | 135573 | 3686 | 3.298 | 0.861 (0.825-0.898) |  | 0.902 (0.864-0.941) |  |
|  |  | <90/85 | 158981 | 5012 | 3.832 | 1 (Ref.) |  | 1 (Ref.) |  |
|  |  | <95/90 | 107035 | 3876 | 4.410 | 1.151 (1.104-1.200) |  | 1.104 (1.059-1.151) |  |
|  |  | <100/95 | 57158 | 2395 | 5.120 | 1.337 (1.274-1.404) |  | 1.233 (1.174-1.295) |  |
|  |  | ≥100/95 | 32567 | 1706 | 6.468 | 1.693 (1.602-1.788) |  | 1.489 (1.409-1.574) |  |
|  |  | p-value |  |  |  | <0.001 |  | <0.001 |  |
| 70- | Male | <80/75 | 73150 | 5491 | 11.008 | 0.971 (0.934-1.009) | 0.426 | 0.992 (0.954-1.032) | 0.482 |
|  |  | <85/80 | 61905 | 4837 | 10.958 | 0.961 (0.923-1.000) |  | 0.976 (0.938-1.016) |  |
|  |  | <90/85 | 58772 | 4834 | 11.418 | 1 (Ref.) |  | 1 (Ref.) |  |
|  |  | <95/90 | 40769 | 3787 | 12.908 | 1.131 (1.084-1.180) |  | 1.110 (1.063-1.158) |  |
|  |  | <100/95 | 17910 | 1884 | 14.753 | 1.295 (1.228-1.366) |  | 1.241 (1.177-1.309) |  |
|  |  | ≥100/95 | 8681 | 999 | 16.449 | 1.449 (1.354-1.551) |  | 1.355 (1.265-1.451) |  |
|  |  | p-value |  |  |  | <0.001 |  | <0.001 |  |
|  | Female | <80/75 | 63197 | 3767 | 7.919 | 0.947 (0.908-0.987) |  | 0.956 (0.917-0.998) |  |
|  |  | <85/80 | 63098 | 3814 | 7.841 | 0.934 (0.896-0.974) |  | 0.949 (0.91-0.99) |  |
|  |  | <90/85 | 80298 | 5224 | 8.400 | 1 (Ref.) |  | 1 (Ref.) |  |
|  |  | <95/90 | 60608 | 4224 | 9.015 | 1.074 (1.031-1.119) |  | 1.053 (1.011-1.097) |  |
|  |  | <100/95 | 36802 | 2881 | 10.183 | 1.214 (1.16-1.271) |  | 1.167 (1.115-1.222) |  |
|  |  | ≥100/95 | 23564 | 2150 | 12.032 | 1.440 (1.37-1.514) |  | 1.337 (1.271-1.407) |  |
|  |  | p-value |  |  |  | <0.001 |  | <0.001 |  |

Abbreviation: WC, waist circumference; M, male; F, female; IR, incidence rate; PY, person-years; HR, hazard ratio; CI, confidence interval.

Model 1 is an unadjusted risk.

Model 3 was adjusted for age, sex, smoking status (never smoker, ex-smoker, or current smoker), alcohol intake (non, mild, or heavy drinker, g/day), regular exercise (performing > 30 min of moderate physical activity ≥ five times a week or > 20 min of vigorous physical activity ≥ three times a week), comorbidities (hypertension, diabetes mellitus, dyslipidemia, myocardial infarction, peripheral artery disease, chronic obstructive pulmonary disease, liver cirrhosis, chronic kidney disease, and cancer), low-income level (income in lower 20% among the entire Korean population thus supported by the medical aid program), and frailty index, Charlson Comorbidity Index (CCI).

Supplementary Table 6. The risk of ischaemic stroke according to body mass index among different age groups.

|  | BMI | Number | Events | IR  (1000PY) | Model 1 HR (95% CI) | Model 2 HR (95% CI) | Model 3 HR (95% CI) |
| --- | --- | --- | --- | --- | --- | --- | --- |
| 20-29 | <18.5 | 131414 | 70 | 0.064 | 1.018 (0.787-1.318) | 1.105 (0.850-1.435) | 1.106 (0.851-1.437) |
|  | <23 | 629174 | 329 | 0.063 | 1 (Ref.) | 1 (Ref.) | 1 (Ref.) |
|  | <25 | 193269 | 136 | 0.085 | 1.347 (1.103-1.645) | 1.215 (0.989-1.491) | 1.177 (0.959-1.446) |
|  | <30 | 191129 | 215 | 0.136 | 2.159 (1.818-2.564) | 1.886 (1.574-2.260) | 1.684 (1.400-2.025) |
|  | ≥30 | 39956 | 99 | 0.300 | 4.780 (3.818-5.984) | 4.265 (3.388-5.369) | 2.996 (2.341-3.836) |
|  | p-value |  |  |  | <0.001 | <0.001 | <0.001 |
| 30-39 | <18.5 | 81204 | 97 | 0.144 | 0.922 (0.748-1.135) | 1.088 (0.882-1.341) | 1.105 (0.896-1.362) |
|  | <23 | 765984 | 993 | 0.156 | 1 (Ref.) | 1 (Ref.) | 1 (Ref.) |
|  | <25 | 421422 | 792 | 0.227 | 1.451 (1.322-1.593) | 1.288 (1.171-1.416) | 1.209 (1.099-1.330) |
|  | <30 | 525545 | 1346 | 0.310 | 1.982 (1.826-2.152) | 1.693 (1.555-1.843) | 1.400 (1.283-1.527) |
|  | ≥30 | 84555 | 401 | 0.576 | 3.689 (3.285-4.143) | 3.260 (2.898-3.667) | 2.055 (1.815-2.328) |
|  | p-value |  |  |  | <0.001 | <0.001 | <0.001 |
| 40-49 | <18.5 | 58200 | 239 | 0.499 | 0.989 (0.868-1.126) | 1.009 (0.886-1.149) | 1.051 (0.923-1.198) |
|  | <23 | 1033176 | 4317 | 0.504 | 1 (Ref.) | 1 (Ref.) | 1 (Ref.) |
|  | <25 | 658174 | 3546 | 0.651 | 1.290 (1.234-1.349) | 1.168 (1.117-1.221) | 1.078 (1.030-1.127) |
|  | <30 | 743641 | 5085 | 0.828 | 1.642 (1.577-1.710) | 1.427 (1.369-1.487) | 1.161 (1.113-1.212) |
|  | ≥30 | 82876 | 720 | 1.056 | 2.095 (1.936-2.267) | 1.958 (1.809-2.120) | 1.253 (1.155-1.359) |
|  | p-value |  |  |  | <0.001 | <0.001 | <0.001 |
| 50-59 | <18.5 | 32671 | 434 | 1.643 | 1.174 (1.066-1.293) | 1.046 (0.950-1.153) | 1.108 (1.006-1.221) |
|  | <23 | 692868 | 8001 | 1.402 | 1 (Ref.) | 1 (Ref.) | 1 (Ref.) |
|  | <25 | 562230 | 7292 | 1.572 | 1.121 (1.086-1.157) | 1.098 (1.064-1.134) | 1.024 (0.992-1.057) |
|  | <30 | 656746 | 9692 | 1.791 | 1.278 (1.240-1.316) | 1.235 (1.199-1.272) | 1.054 (1.023-1.087) |
|  | ≥30 | 64221 | 1112 | 2.106 | 1.504 (1.412-1.601) | 1.604 (1.506-1.708) | 1.164 (1.092-1.241) |
|  | p-value |  |  |  | <0.001 | <0.001 | <0.001 |
| 60-69 | <18.5 | 24448 | 850 | 4.547 | 1.125 (1.050-1.206) | 0.984 (0.918-1.055) | 1.056 (0.985-1.132) |
|  | <23 | 379612 | 12368 | 4.059 | 1 (Ref.) | 1 (Ref.) | 1 (Ref.) |
|  | <25 | 331487 | 10712 | 3.986 | 0.981 (0.956-1.007) | 1.042 (1.016-1.070) | 0.979 (0.953-1.004) |
|  | <30 | 418028 | 14168 | 4.173 | 1.027 (1.003-1.052) | 1.131 (1.104-1.159) | 0.998 (0.973-1.023) |
|  | ≥30 | 41548 | 1546 | 4.592 | 1.131 (1.073-1.192) | 1.346 (1.276-1.420) | 1.071 (1.015-1.130) |
|  | p-value |  |  |  | <0.001 | <0.001 | <0.001 |
| 70- | <18.5 | 28494 | 1920 | 10.536 | 1.026 (0.979-1.076) | 0.925 (0.882-0.969) | 0.971 (0.925-1.018) |
|  | <23 | 222453 | 16668 | 10.361 | 1 (Ref.) | 1 (Ref.) | 1 (Ref.) |
|  | <25 | 146864 | 11019 | 9.966 | 0.959 (0.936-0.982) | 1.026 (1.002-1.052) | 0.980 (0.956-1.004) |
|  | <30 | 173364 | 12977 | 9.819 | 0.944 (0.923-0.966) | 1.058 (1.033-1.082) | 0.972 (0.949-0.996) |
|  | ≥30 | 17579 | 1357 | 10.114 | 0.972 (0.920-1.027) | 1.149 (1.087-1.215) | 1.001 (0.947-1.059) |
|  | p-value |  |  |  | <0.001 | <0.001 | <0.001 |
| P for interaction | |  |  |  | <0.001 | <0.001 | <0.001 |

Abbreviation: BMI, body mass index; IR, incidence rate; PY, person-years; HR, hazard ratio; CI, confidence interval.

Model 1 is an unadjusted risk.

Model 2 was adjusted for age, sex, smoking status (never smoker, ex-smoker, or current smoker), alcohol intake (non, mild, or heavy drinker, g/day), regular exercise (performing > 30 min of moderate physical activity ≥ five times a week or > 20 min of vigorous physical activity ≥ three times a week), and low-income level (income in lower 20% among the entire Korean population thus supported by the medical aid program).

Model 3 was adjusted for comorbidities (hypertension, diabetes mellitus, dyslipidemia, myocardial infarction, peripheral artery disease, chronic obstructive pulmonary disease, liver cirrhosis, chronic kidney disease, and cancer), and frailty index, Charlson Comorbidity Index (CCI), in addition to Model 2.

Supplementary Table 7. The risk of ischaemic stroke according to waist circumference among different age groups.

| Age | WC (M/F) | Number | Events | IR  (1000PY) | Model 1 HR (95% CI) | Model 2 HR (95% CI) | Model 3 HR (95% CI) |
| --- | --- | --- | --- | --- | --- | --- | --- |
| 20-29 | <80/75 | 759354 | 412 | 0.065 | 0.576 (0.466-0.711) | 0.674 (0.541-0.840) | 0.737 (0.590-0.920) |
|  | <85/80 | 203556 | 154 | 0.091 | 0.804 (0.628-1.028) | 0.825 (0.645-1.055) | 0.869 (0.679-1.112) |
|  | <90/85 | 114921 | 108 | 0.113 | 1 (Ref.) | 1 (Ref.) | 1 (Ref.) |
|  | <95/90 | 57784 | 67 | 0.140 | 1.235 (0.911-1.676) | 1.222(0.901,1.658) | 1.134 (0.835-1.539) |
|  | <100/95 | 27223 | 53 | 0.235 | 2.076 (1.495-2.884) | 2.068(1.488,2.873) | 1.752 (1.258-2.44) |
|  | ≥100/95 | 22104 | 55 | 0.301 | 2.664 (1.925-3.685) | 2.695(1.947,3.73) | 1.940 (1.390-2.707) |
|  | p-value |  |  |  | <0.001 | <0.001 | <0.001 |
| 30-39 | <80/75 | 812481 | 1058 | 0.157 | 0.577 (0.525-0.634) | 0.690 (0.626-0.760) | 0.796 (0.721-0.878) |
|  | <85/80 | 450044 | 880 | 0.236 | 0.867 (0.786-0.957) | 0.898 (0.813-0.991) | 0.970 (0.878-1.070) |
|  | <90/85 | 319693 | 720 | 0.272 | 1 (Ref.) | 1 (Ref.) | 1 (Ref.) |
|  | <95/90 | 173521 | 471 | 0.328 | 1.207 (1.075-1.356) | 1.186 (1.056-1.332) | 1.080 (0.961-1.213) |
|  | <100/95 | 75640 | 258 | 0.413 | 1.519 (1.318-1.751) | 1.506 (1.306-1.736) | 1.239 (1.074-1.430) |
|  | ≥100/95 | 47331 | 242 | 0.621 | 2.285 (1.975-2.643) | 2.364 (2.043-2.735) | 1.634 (1.408-1.897) |
|  | p-value |  |  |  | <0.001 | <0.001 | <0.001 |
| 40-49 | <80/75 | 1009746 | 3859 | 0.461 | 0.600 (0.572-0.629) | 0.725 (0.691-0.760) | 0.849 (0.809-0.892) |
|  | <85/80 | 657560 | 3657 | 0.672 | 0.875 (0.834-0.917) | 0.914 (0.872-0.959) | 0.988 (0.942-1.036) |
|  | <90/85 | 496234 | 3152 | 0.769 | 1 (Ref.) | 1 (Ref.) | 1 (Ref.) |
|  | <95/90 | 259688 | 1899 | 0.886 | 1.153 (1.089-1.221) | 1.120 (1.058-1.185) | 1.025 (0.968-1.085) |
|  | <100/95 | 102164 | 845 | 1.003 | 1.306 (1.211-1.409) | 1.275 (1.182-1.376) | 1.059 (0.981-1.143) |
|  | ≥100/95 | 50675 | 495 | 1.188 | 1.547 (1.407-1.701) | 1.576 (1.433-1.733) | 1.134 (1.030-1.248) |
|  | p-value |  |  |  | <0.001 | <0.001 | <0.001 |
| 50-59 | <80/75 | 572883 | 5863 | 1.241 | 0.736 (0.711-0.763) | 0.824 (0.795-0.854) | 0.936 (0.903-0.970) |
|  | <85/80 | 527734 | 6675 | 1.534 | 0.911 (0.880-0.942) | 0.931 (0.900-0.963) | 0.986 (0.953-1.020) |
|  | <90/85 | 470867 | 6537 | 1.685 | 1 (Ref.) | 1 (Ref.) | 1 (Ref.) |
|  | <95/90 | 270789 | 4409 | 1.979 | 1.175 (1.131-1.221) | 1.142 (1.100-1.187) | 1.075 (1.035-1.117) |
|  | <100/95 | 112482 | 1984 | 2.146 | 1.274 (1.212-1.340) | 1.259 (1.197-1.323) | 1.112 (1.058-1.170) |
|  | ≥100/95 | 53981 | 1063 | 2.403 | 1.428 (1.338-1.523) | 1.467 (1.375-1.565) | 1.182 (1.107-1.261) |
|  | p-value |  |  |  | <0.001 | <0.001 | <0.001 |
| 60-69 | <80/75 | 253884 | 7398 | 3.627 | 0.897 (0.870-0.924) | 0.853 (0.828-0.879) | 0.954 (0.925-0.983) |
|  | <85/80 | 282834 | 8983 | 3.930 | 0.971 (0.944-0.999) | 0.949 (0.923-0.977) | 0.995 (0.967-1.024) |
|  | <90/85 | 305944 | 10036 | 4.047 | 1 (Ref.) | 1 (Ref.) | 1 (Ref.) |
|  | <95/90 | 204903 | 7316 | 4.414 | 1.091 (1.058-1.124) | 1.086 (1.054-1.119) | 1.040 (1.009-1.072) |
|  | <100/95 | 97556 | 3736 | 4.737 | 1.171 (1.128-1.216) | 1.190 (1.146-1.236) | 1.093 (1.052-1.135) |
|  | ≥100/95 | 50002 | 2175 | 5.416 | 1.341 (1.280-1.404) | 1.385 (1.322-1.451) | 1.194 (1.140-1.252) |
|  | p-value |  |  |  | <0.001 | <0.001 | <0.001 |
| 70- | <80/75 | 136347 | 9626 | 9.910 | 1.009 (0.981-1.037) | 0.922 (0.896-0.948) | 0.993 (0.965-1.021) |
|  | <85/80 | 125003 | 9211 | 9.958 | 1.011 (0.983-1.040) | 0.978 (0.951-1.006) | 1.009 (0.981-1.037) |
|  | <90/85 | 139070 | 10284 | 9.860 | 1 (Ref.) | 1 (Ref.) | 1 (Ref.) |
|  | <95/90 | 101377 | 7776 | 10.205 | 1.035 (1.005-1.066) | 1.046 (1.015-1.077) | 1.015 (0.986-1.046) |
|  | <100/95 | 54712 | 4391 | 10.686 | 1.084 (1.046-1.123) | 1.112 (1.074-1.153) | 1.055 (1.018-1.093) |
|  | ≥100/95 | 32245 | 2653 | 11.045 | 1.121 (1.074-1.170) | 1.157 (1.108-1.208) | 1.061 (1.017-1.108) |
|  | p-value |  |  |  | <0.001 | <0.001 | <0.001 |
| p for interaction | |  |  |  | <0.001 | <0.001 | <0.001 |

Abbreviation: WC, waist circumference; M, male; F, female; IR, incidence rate; PY, person-years; HR, hazard ratio; CI, confidence interval.

Model 1 is an unadjusted risk.

Model 2 was adjusted for age, sex, smoking status (never smoker, ex-smoker, or current smoker), alcohol intake (non, mild, or heavy drinker, g/day), regular exercise (performing > 30 min of moderate physical activity ≥ five times a week or > 20 min of vigorous physical activity ≥ three times a week), and low-income level (income in lower 20% among the entire Korean population thus supported by the medical aid program).

Model 3 was adjusted for comorbidities (hypertension, diabetes mellitus, dyslipidemia, myocardial infarction, peripheral artery disease, chronic obstructive pulmonary disease, liver cirrhosis, chronic kidney disease, and cancer), and frailty index, Charlson Comorbidity Index (CCI), in addition to Model 2.

Supplementary Table 8. The association between body mass index and the risk of ischaemic stroke according to age differentiated by sex.

| Age | Sex | BMI | Number | Event | IR  (1000PY) | Model 1 | p for  interaction | Model 3 | p for  interaction |
| --- | --- | --- | --- | --- | --- | --- | --- | --- | --- |
| 20-29 | Male | <18.5 | 22155 | 17 | 0.092 | 1.386 (0.839-2.291) | 0.220 | 1.449 (0.876-2.398) | 0.385 |
|  |  | <23 | 261354 | 145 | 0.067 | 1 (Ref.) |  | 1 (Ref.) |  |
|  |  | <25 | 139549 | 110 | 0.095 | 1.423 (1.110-1.823) |  | 1.324 (1.032-1.698) |  |
|  |  | <30 | 150415 | 183 | 0.147 | 2.202 (1.771-2.738) |  | 1.860 (1.488-2.326) |  |
|  |  | ≥30 | 29273 | 82 | 0.339 | 5.097 (3.888-6.682) |  | 3.480 (2.604-4.649) |  |
|  |  | p-value |  |  |  | <0.001 |  | <0.001 |  |
|  | Female | <18.5 | 109259 | 53 | 0.058 | 0.968 (0.713,-1.314) |  | 0.976 (0.719-1.326) |  |
|  |  | <23 | 367820 | 184 | 0.060 | 1 (Ref.) |  | 1 (Ref.) |  |
|  |  | <25 | 53720 | 26 | 0.058 | 0.969 (0.643-1.462) |  | 0.930 (0.616-1.404) |  |
|  |  | <30 | 40714 | 32 | 0.095 | 1.577 (1.083-2.295) |  | 1.376 (0.938-2.019) |  |
|  |  | ≥30 | 10683 | 17 | 0.192 | 3.200 (1.947-5.259) |  | 2.030 (1.184-3.481) |  |
|  |  | p-value |  |  |  | <0.001 |  | 0.0603 |  |
| 30-39 | Male | <18.5 | 28273 | 56 | 0.239 | 1.256 (0.957-1.648) | 0.000 | 1.297 (0.988-1.703) | 0.001 |
|  |  | <23 | 453456 | 717 | 0.190 | 1 (Ref.) |  | 1 (Ref.) |  |
|  |  | <25 | 347683 | 671 | 0.233 | 1.222 (1.100-1.357) |  | 1.125 (1.012-1.251) |  |
|  |  | <30 | 464738 | 1227 | 0.319 | 1.676 (1.528-1.838) |  | 1.335 (1.214-1.467) |  |
|  |  | ≥30 | 71769 | 365 | 0.617 | 3.246 (2.862-3.682) |  | 2.004 (1.753-2.29) |  |
|  |  | p-value |  |  |  | <0.001 |  | <0.001 |  |
|  | Female | <18.5 | 52931 | 41 | 0.093 | 0.876 (0.631-1.216) |  | 0.972 (0.699-1.351) |  |
|  |  | <23 | 312528 | 276 | 0.106 | 1 (Ref.) |  | 1 (Ref.) |  |
|  |  | <25 | 73739 | 121 | 0.198 | 1.863 (1.505-2.307) |  | 1.657 (1.336-2.055) |  |
|  |  | <30 | 60807 | 119 | 0.237 | 2.225 (1.795-2.759) |  | 1.759 (1.409-2.196) |  |
|  |  | ≥30 | 12786 | 36 | 0.341 | 3.207 (2.266-4.539) |  | 1.976 (1.367-2.857) |  |
|  |  | p-value |  |  |  | <0.001 |  | <0.001 |  |
| 40-49 | Male | <18.5 | 21116 | 147 | 0.854 | 1.108 (0.939-1.309) | <0.001 | 1.078 (0.913-1.273) | <0.001 |
|  |  | <23 | 422685 | 2690 | 0.772 | 1 (Ref.) |  | 1 (Ref.) |  |
|  |  | <25 | 383071 | 2471 | 0.781 | 1.012 (0.958-1.069) |  | 0.968 (0.916-1.022) |  |
|  |  | <30 | 496090 | 3862 | 0.944 | 1.224 (1.165-1.286) |  | 1.048 (0.996-1.102) |  |
|  |  | ≥30 | 47251 | 470 | 1.213 | 1.574 (1.427-1.736) |  | 1.088 (0.984-1.203) |  |
|  |  | p-value |  |  |  | <0.001 |  | 0.015 |  |
|  | Female | <18.5 | 37084 | 92 | 0.299 | 0.934 (0.757-1.152) |  | 1.000 (0.810-1.234) |  |
|  |  | <23 | 610491 | 1627 | 0.321 | 1 (Ref.) |  | 1 (Ref.) |  |
|  |  | <25 | 275103 | 1075 | 0.471 | 1.467 (1.358-1.585) |  | 1.306 (1.208-1.411) |  |
|  |  | <30 | 247551 | 1223 | 0.596 | 1.858 (1.725-2.001) |  | 1.426 (1.320-1.539) |  |
|  |  | ≥30 | 35625 | 250 | 0.849 | 2.648 (2.318-3.025) |  | 1.579 (1.374-1.813) |  |
|  |  | p-value |  |  |  | <0.001 |  | <0.001 |  |
| 50-59 | Male | <18.5 | 15769 | 318 | 2.559 | 1.195 (1.067-1.338) | <0.001 | 1.126 (1.005-1.261) | <0.001 |
|  |  | <23 | 304282 | 5314 | 2.148 | 1 (Ref.) |  | 1 (Ref.) |  |
|  |  | <25 | 288030 | 4932 | 2.093 | 0.974 (0.937-1.012) |  | 0.973 (0.936-1.011) |  |
|  |  | <30 | 358778 | 6555 | 2.234 | 1.040 (1.003-1.078) |  | 0.980 (0.944-1.017) |  |
|  |  | ≥30 | 25153 | 542 | 2.651 | 1.234 (1.130-1.348) |  | 1.004 (0.918-1.098) |  |
|  |  | p-value |  |  |  | <0.001 |  | 0.101 |  |
|  | Female | <18.5 | 16902 | 116 | 0.829 | 0.999 (0.829-1.203) |  | 1.031 (0.856-1.242) |  |
|  |  | <23 | 388586 | 2687 | 0.831 | 1 (Ref.) |  | 1 (Ref.) |  |
|  |  | <25 | 274200 | 2360 | 1.034 | 1.244 (1.177-1.315) |  | 1.126 (1.065-1.191) |  |
|  |  | <30 | 297968 | 3137 | 1.266 | 1.523 (1.447-1.604) |  | 1.211 (1.149-1.277) |  |
|  |  | ≥30 | 39068 | 570 | 1.762 | 2.121 (1.937-2.321) |  | 1.376 (1.254-1.510) |  |
|  |  | p-value |  |  |  | <0.001 |  | <0.001 |  |
| 60-69 | Male | <18.5 | 14401 | 572 | 5.406 | 1.051 (0.965-1.144) | <0.001 | 1.003 (0.921-1.092) | <0.001 |
|  |  | <23 | 198003 | 8035 | 5.177 | 1 (Ref.) |  | 1 (Ref.) |  |
|  |  | <25 | 165615 | 6551 | 4.966 | 0.958 (0.927-0.990) |  | 0.964 (0.933-0.997) |  |
|  |  | <30 | 190746 | 7637 | 5.013 | 0.967 (0.937-0.998) |  | 0.949 (0.919-0.980) |  |
|  |  | ≥30 | 11604 | 539 | 5.881 | 1.136 (1.041-1.239) |  | 1.030 (0.943-1.125) |  |
|  |  | p-value |  |  |  | <0.001 |  | 0.012 |  |
|  | Female | <18.5 | 10047 | 278 | 3.426 | 1.184 (1.049-1.337) |  | 1.170 (1.036-1.322) |  |
|  |  | <23 | 181609 | 4333 | 2.898 | 1 (Ref.) |  | 1 (Ref.) |  |
|  |  | <25 | 165872 | 4161 | 3.041 | 1.049 (1.005-1.095) |  | 1.004 (0.962-1.047) |  |
|  |  | <30 | 227282 | 6531 | 3.489 | 1.204 (1.159-1.251) |  | 1.065 (1.024-1.107) |  |
|  |  | ≥30 | 29944 | 1007 | 4.110 | 1.420 (1.326-1.521) |  | 1.103 (1.029-1.183) |  |
|  |  | p-value |  |  |  | <0.001 |  | <0.001 |  |
| 70- | Male | <18.5 | 14630 | 1007 | 11.503 | 0.996 (0.933-1.063) | 0.603 | 0.954 (0.894-1.019) | 0.822 |
|  |  | <23 | 110723 | 8935 | 11.663 | 1 (Ref.) |  | 1 (Ref.) |  |
|  |  | <25 | 67372 | 5477 | 11.193 | 0.956 (0.925-0.989) |  | 0.969 (0.937-1.003) |  |
|  |  | <30 | 64898 | 5367 | 11.259 | 0.961 (0.929-0.994) |  | 0.966 (0.933-1.000) |  |
|  |  | ≥30 | 3564 | 326 | 12.597 | 1.076 (0.964-1.202) |  | 1.030 (0.922-1.151) |  |
|  |  | p-value |  |  |  | 0.017 |  | 0.149 |  |
|  | Female | <18.5 | 13864 | 913 | 9.642 | 1.058 (0.988-1.133) |  | 0.988 (0.922-1.059) |  |
|  |  | <23 | 111730 | 7733 | 9.178 | 1 (Ref.) |  | 1 (Ref.) |  |
|  |  | <25 | 79492 | 5542 | 8.992 | 0.978 (0.945-1.012) |  | 0.991 (0.957-1.026) |  |
|  |  | <30 | 108466 | 7610 | 9.007 | 0.979 (0.948-1.010) |  | 0.979 (0.947-1.011) |  |
|  |  | ≥30 | 14015 | 1031 | 9.521 | 1.036 (0.970-1.105) |  | 0.997 (0.933-1.065) |  |
|  |  | p-value |  |  |  | 0.07 |  | 0.775 |  |

Abbreviation: WC, waist circumference; IR, incidence rate; PY, person-years; HR, hazard ratio; CI, confidence interval.

Model 1 is an unadjusted risk.

Model 3 was adjusted for age, sex, smoking status (never smoker, ex-smoker, or current smoker), alcohol intake (non, mild, or heavy drinker, g/day), regular exercise (performing > 30 min of moderate physical activity ≥ five times a week or > 20 min of vigorous physical activity ≥ three times a week), comorbidities (hypertension, diabetes mellitus, dyslipidemia, myocardial infarction, peripheral artery disease, chronic obstructive pulmonary disease, liver cirrhosis, chronic kidney disease, and cancer), low-income level (income in lower 20% among the entire Korean population thus supported by the medical aid program), and frailty index, Charlson Comorbidity Index (CCI).

Supplementary Table 9. The association between waist circumference and the risk of ischaemic stroke according to age differentiated by sex.

| Age | Sex | WC (M/F) | Number | Event | IR  (1000PY) | Model 1 | p for  interaction | Model 3 | p for  interaction |
| --- | --- | --- | --- | --- | --- | --- | --- | --- | --- |
| 20-29 | Male | <80/75 | 287581 | 181 | 0.076 | 0.662 (0.510-0.858) | 0.105 | 0.774 (0.595-1.008) | 0.124 |
|  |  | <85/80 | 145984 | 125 | 0.103 | 0.902 (0.683-1.190) |  | 0.965 (0.731-1.275) |  |
|  |  | <90/85 | 87530 | 83 | 0.114 | 1 (Ref.) |  | 1 (Ref.) |  |
|  |  | <95/90 | 44969 | 54 | 0.145 | 1.268 (0.900-1.787) |  | 1.174 (0.833-1.655) |  |
|  |  | <100/95 | 20669 | 43 | 0.251 | 2.199 (1.522-3.178) |  | 1.873 (1.292-2.715) |  |
|  |  | ≥100/95 | 16013 | 51 | 0.386 | 3.383 (2.387-4.795) |  | 2.521 (1.762-3.608) |  |
|  |  | p-value |  |  |  | <0.001 |  | <0.001 |  |
|  | Female | <80/75 | 471773 | 231 | 0.059 | 0.534 (0.354-0.807) |  | 0.602 (0.397-0.913) |  |
|  |  | <85/80 | 57572 | 29 | 0.061 | 0.551 (0.323-0.941) |  | 0.586 (0.343-1.002) |  |
|  |  | <90/85 | 27391 | 25 | 0.110 | 1 (Ref.) |  | 1 (Ref.) |  |
|  |  | <95/90 | 12815 | 13 | 0.122 | 1.111 (0.568-2.172) |  | 1.020 (0.521-1.996) |  |
|  |  | <100/95 | 6554 | 10 | 0.184 | 1.674 (0.804-3.485) |  | 1.365 (0.652-2.861) |  |
|  |  | ≥100/95 | 6091 | 4 | 0.079 | 0.720 (0.250-2.068) |  | 0.461 (0.158-1.346) |  |
|  |  | p-value |  |  |  | <0.001 |  | 0.015 |  |
| 30-39 | Male | <80/75 | 457870 | 741 | 0.195 | 0.692 (0.623-0.769) | 0.124 | 0.839 (0.754-0.934) | 0.088 |
|  |  | <85/80 | 372054 | 765 | 0.248 | 0.880 (0.792-0.977) |  | 0.970 (0.873-1.077) |  |
|  |  | <90/85 | 277241 | 647 | 0.282 | 1 (Ref.) |  | 1 (Ref.) |  |
|  |  | <95/90 | 153723 | 432 | 0.340 | 1.206 (1.068-1.362) |  | 1.085 (0.961-1.226) |  |
|  |  | <100/95 | 65821 | 233 | 0.428 | 1.522 (1.310-1.767) |  | 1.238 (1.064-1.439) |  |
|  |  | ≥100/95 | 39210 | 218 | 0.675 | 2.400 (2.058-2.798) |  | 1.672 (1.429-1.956) |  |
|  |  | p-value |  |  |  | <0.001 |  | <0.001 |  |
|  | Female | <80/75 | 354611 | 317 | 0.108 | 0.518 (0.401-0.668) |  | 0.629 (0.486-0.814) |  |
|  |  | <85/80 | 77990 | 115 | 0.178 | 0.856 (0.639-1.148) |  | 0.924 (0.689-1.240) |  |
|  |  | <90/85 | 42452 | 73 | 0.208 | 1 (Ref.) |  | 1 (Ref.) |  |
|  |  | <95/90 | 19798 | 39 | 0.238 | 1.146 (0.777-1.690) |  | 1.062 (0.719-1.568) |  |
|  |  | <100/95 | 9819 | 25 | 0.308 | 1.484 (0.942-2.337) |  | 1.266 (0.802-1.999) |  |
|  |  | ≥100/95 | 8121 | 24 | 0.358 | 1.722 (1.086-2.731) |  | 1.289 (0.805-2.066) |  |
|  |  | p-value |  |  |  | <0.001 |  | <0.001 |  |
| 40-49 | Male | <80/75 | 373046 | 2187 | 0.711 | 0.803 (0.758-0.851) | <0.001 | 0.940 (0.886-0.997) | <0.001 |
|  |  | <85/80 | 389608 | 2641 | 0.821 | 0.928 (0.878-0.981) |  | 1.008 (0.953-1.065) |  |
|  |  | <90/85 | 326229 | 2382 | 0.885 | 1 (Ref.) |  | 1 (Ref.) |  |
|  |  | <95/90 | 181005 | 1469 | 0.986 | 1.114 (1.043-1.188) |  | 1.011 (0.947-1.079) |  |
|  |  | <100/95 | 69248 | 616 | 1.082 | 1.222 (1.119-1.336) |  | 1.005 (0.920-1.099) |  |
|  |  | ≥100/95 | 31077 | 345 | 1.355 | 1.532 (1.368-1.715) |  | 1.107 (0.987-1.240) |  |
|  |  | p-value |  |  |  | <0.001 |  | 0.047 |  |
|  | Female | <80/75 | 636700 | 1672 | 0.316 | 0.579 (0.532-0.631) |  | 0.722 (0.662-0.787) |  |
|  |  | <85/80 | 267952 | 1016 | 0.457 | 0.837 (0.762-0.919) |  | 0.925 (0.842-1.016) |  |
|  |  | <90/85 | 170005 | 770 | 0.546 | 1 (Ref.) |  | 1 (Ref.) |  |
|  |  | <95/90 | 78683 | 430 | 0.659 | 1.207 (1.073-1.358) |  | 1.085 (0.964-1.221) |  |
|  |  | <100/95 | 32916 | 229 | 0.840 | 1.540 (1.329-1.785) |  | 1.234 (1.064-1.432) |  |
|  |  | ≥100/95 | 19598 | 150 | 0.926 | 1.698 (1.426-2.023) |  | 1.154 (0.967-1.378) |  |
|  |  | p-value |  |  |  | <0.001 |  | <0.001 |  |
| 50-59 | Male | <80/75 | 222646 | 3579 | 1.977 | 0.915 (0.875-0.955) | <0.001 | 0.988 (0.945-1.033) | <0.001 |
|  |  | <85/80 | 273519 | 4624 | 2.069 | 0.957 (0.919-0.997) |  | 1.001 (0.961-1.043) |  |
|  |  | <90/85 | 257398 | 4549 | 2.162 | 1 (Ref.) |  | 1 (Ref.) |  |
|  |  | <95/90 | 154978 | 3016 | 2.384 | 1.103 (1.053-1.155) |  | 1.033 (0.986-1.081) |  |
|  |  | <100/95 | 59342 | 1326 | 2.745 | 1.270 (1.195-1.350) |  | 1.111 (1.045-1.182) |  |
|  |  | ≥100/95 | 24129 | 567 | 2.899 | 1.343 (1.231-1.465) |  | 1.061 (0.972-1.158) |  |
|  |  | p-value |  |  |  | <0.001 |  | 0.006 |  |
|  | Female | <80/75 | 350237 | 2284 | 0.784 | 0.700 (0.659-0.744) |  | 0.864 (0.813-0.919) |  |
|  |  | <85/80 | 254215 | 2051 | 0.969 | 0.866 (0.814-0.921) |  | 0.953 (0.896-1.014) |  |
|  |  | <90/85 | 213469 | 1988 | 1.119 | 1 (Ref.) |  | 1 (Ref.) |  |
|  |  | <95/90 | 115811 | 1393 | 1.447 | 1.293 (1.208-1.385) |  | 1.181 (1.102-1.265) |  |
|  |  | <100/95 | 53140 | 658 | 1.491 | 1.332 (1.220-1.455) |  | 1.106 (1.012-1.208) |  |
|  |  | ≥100/95 | 29852 | 496 | 2.011 | 1.798 (1.630-1.984) |  | 1.329 (1.204-1.469) |  |
|  |  | p-value |  |  |  | <0.001 |  | <0.001 |  |
| 60-69 | Male | <80/75 | 130444 | 4838 | 4.734 | 0.957 (0.921-0.994) | <0.001 | 0.997 (0.959-1.037) | <0.001 |
|  |  | <85/80 | 147261 | 5811 | 4.978 | 1.005 (0.969-1.042) |  | 1.032 (0.995-1.071) |  |
|  |  | <90/85 | 146963 | 5797 | 4.955 | 1 (Ref.) |  | 1 (Ref.) |  |
|  |  | <95/90 | 97868 | 4159 | 5.354 | 1.081 (1.039-1.125) |  | 1.040 (0.999-1.082) |  |
|  |  | <100/95 | 40398 | 1826 | 5.712 | 1.153 (1.094-1.216) |  | 1.065 (1.010-1.122) |  |
|  |  | ≥100/95 | 17435 | 903 | 6.620 | 1.339 (1.249-1.436) |  | 1.151 (1.073-1.235) |  |
|  |  | p-value |  |  |  | <0.001 |  | 0.001 |  |
|  | Female | <80/75 | 123440 | 2560 | 2.516 | 0.777 (0.740-0.816) |  | 0.891 (0.848-0.936) |  |
|  |  | <85/80 | 135573 | 3172 | 2.836 | 0.876 (0.837-0.917) |  | 0.939 (0.897-0.983) |  |
|  |  | <90/85 | 158981 | 4239 | 3.236 | 1 (Ref.) |  | 1 (Ref.) |  |
|  |  | <95/90 | 107035 | 3157 | 3.584 | 1.108 (1.058-1.160) |  | 1.036 (0.990-1.085) |  |
|  |  | <100/95 | 57158 | 1910 | 4.072 | 1.259 (1.193-1.329) |  | 1.107 (1.048-1.169) |  |
|  |  | ≥100/95 | 32567 | 1272 | 4.797 | 1.485 (1.395-1.581) |  | 1.197 (1.124-1.275) |  |
|  |  | p-value |  |  |  | <0.001 |  | <0.001 |  |
| 70- | Male | <80/75 | 73150 | 5513 | 11.082 | 0.980 (0.943-1.019) | 0.201 | 0.988 (0.950-1.028) | 0.266 |
|  |  | <85/80 | 61905 | 4990 | 11.320 | 0.998 (0.960-1.039) |  | 1.011 (0.972-1.052) |  |
|  |  | <90/85 | 58772 | 4800 | 11.349 | 1 (Ref.) |  | 1 (Ref.) |  |
|  |  | <95/90 | 40769 | 3373 | 11.443 | 1.008 (0.965-1.053) |  | 0.984 (0.941-1.028) |  |
|  |  | <100/95 | 17910 | 1645 | 12.826 | 1.131 (1.069-1.196) |  | 1.074 (1.015-1.136) |  |
|  |  | ≥100/95 | 8681 | 791 | 12.913 | 1.139 (1.057-1.228) |  | 1.039 (0.963-1.120) |  |
|  |  | p-value |  |  |  | <0.001 |  | 0.048 |  |
|  | Female | <80/75 | 63197 | 4113 | 8.680 | 0.984 (0.945-1.024) |  | 0.997 (0.957-1.038) |  |
|  |  | <85/80 | 63098 | 4221 | 8.719 | 0.986 (0.947-1.026) |  | 1.004 (0.964-1.045) |  |
|  |  | <90/85 | 80298 | 5484 | 8.845 | 1 (Ref.) |  | 1 (Ref.) |  |
|  |  | <95/90 | 60608 | 4403 | 9.425 | 1.066 (1.024-1.109) |  | 1.040 (1.000-1.082) |  |
|  |  | <100/95 | 36802 | 2746 | 9.715 | 1.099 (1.050-1.150) |  | 1.044 (0.998-1.094) |  |
|  |  | ≥100/95 | 23564 | 1862 | 10.406 | 1.178 (1.118-1.242) |  | 1.073 (1.017-1.131) |  |
|  |  | p-value |  |  |  | <0.001 |  | 0.027 |  |

Abbreviation: WC, waist circumference; M, male; F, female; IR, incidence rate; PY, person-years; HR, hazard ratio; CI, confidence interval.

Model 1 is an unadjusted risk.

Model 3 was adjusted for age, sex, smoking status (never smoker, ex-smoker, or current smoker), alcohol intake (non, mild, or heavy drinker, g/day), regular exercise (performing > 30 min of moderate physical activity ≥ five times a week or > 20 min of vigorous physical activity ≥ three times a week), comorbidities (hypertension, diabetes mellitus, dyslipidemia, myocardial infarction, peripheral artery disease, chronic obstructive pulmonary disease, liver cirrhosis, chronic kidney disease, and cancer), low-income level (income in lower 20% among the entire Korean population thus supported by the medical aid program), and frailty index, Charlson Comorbidity Index (CCI).

**Figures**

Supplementary Figure 1. A) The distribution of BMI and according to age, B) The distribution of sex at each BMI according to age

Abbreviation: BMI, body mass index.


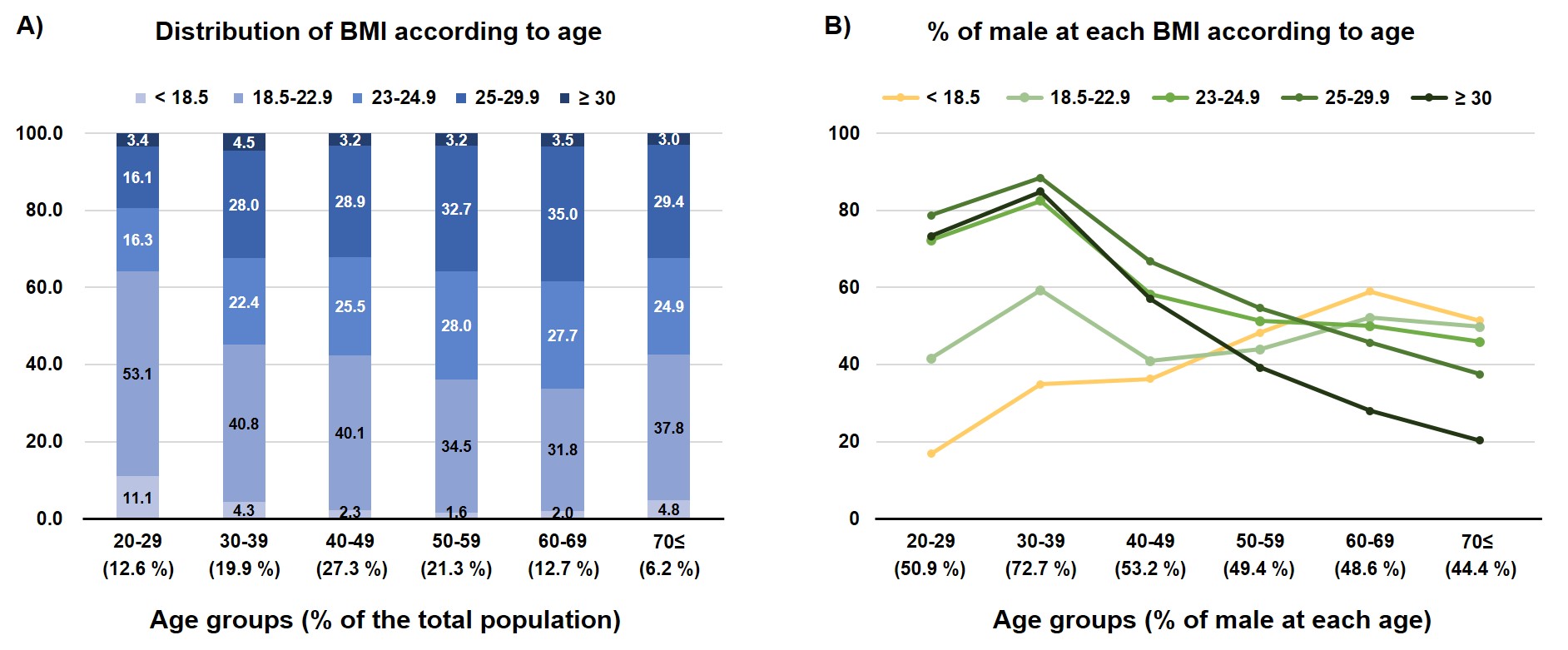


Supplementary Figure 2. A) The BMI distribution of current smokers, B) proportion of current smokers at each BMI according to age

Abbreviation: BMI, body mass index.


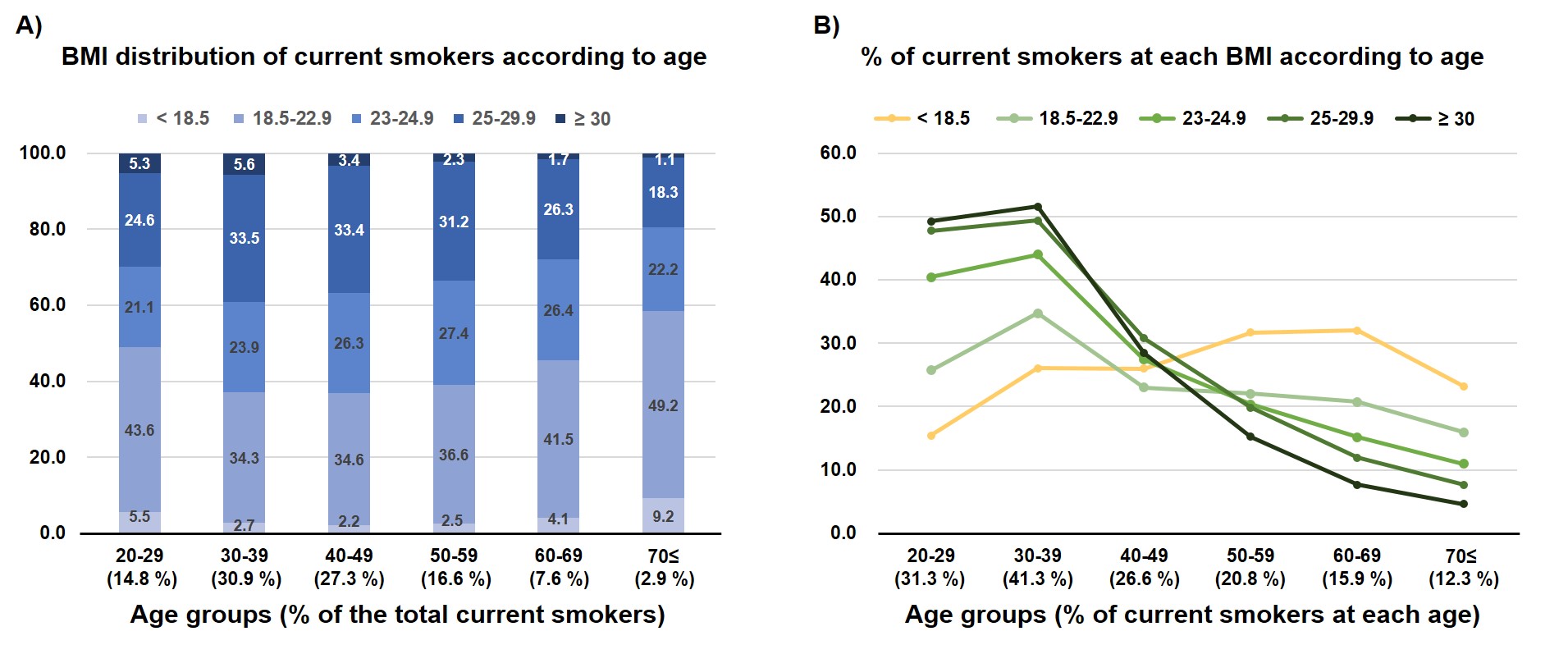


Supplementary Figure 3. A) The BMI distribution of heavy drinkers, B) proportion of heavy drinkers at each BMI according to age

Abbreviation: BMI, body mass index.


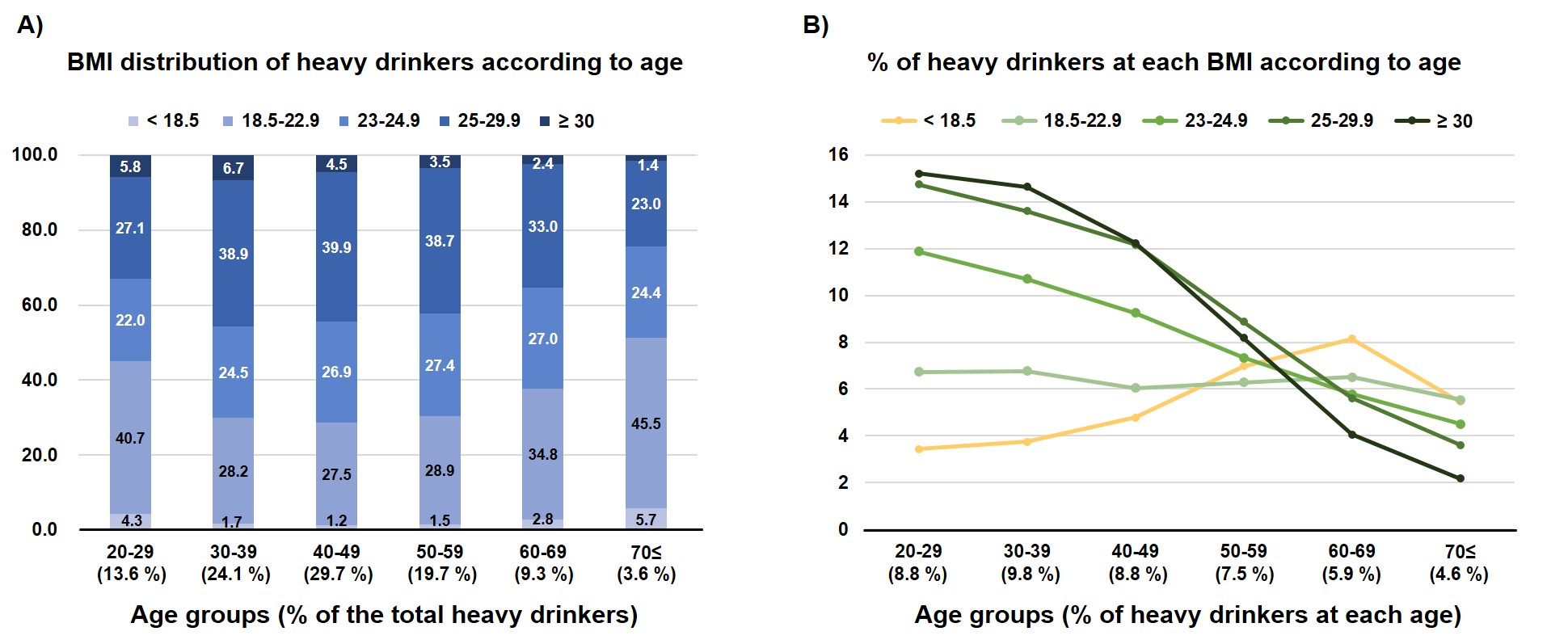


Supplementary Figure 4. A) The BMI distribution of regular exercisers, B) proportion of regular exercisers at each BMI according to age

Abbreviation: BMI, body mass index.


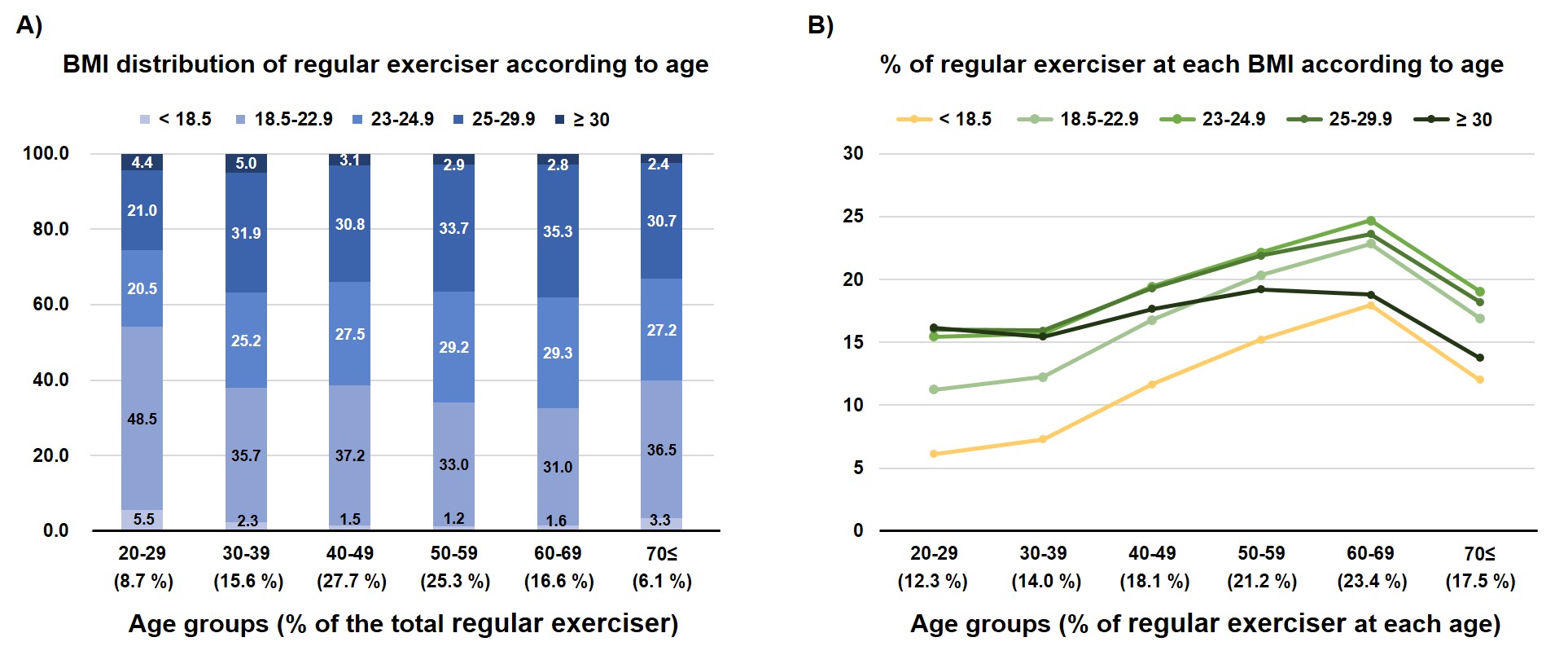


Supplementary Figure 5. A) The BMI distribution of diabetes mellitus, B) proportion of diabetes mellitus at each BMI according to age

Abbreviation: BMI, body mass index.


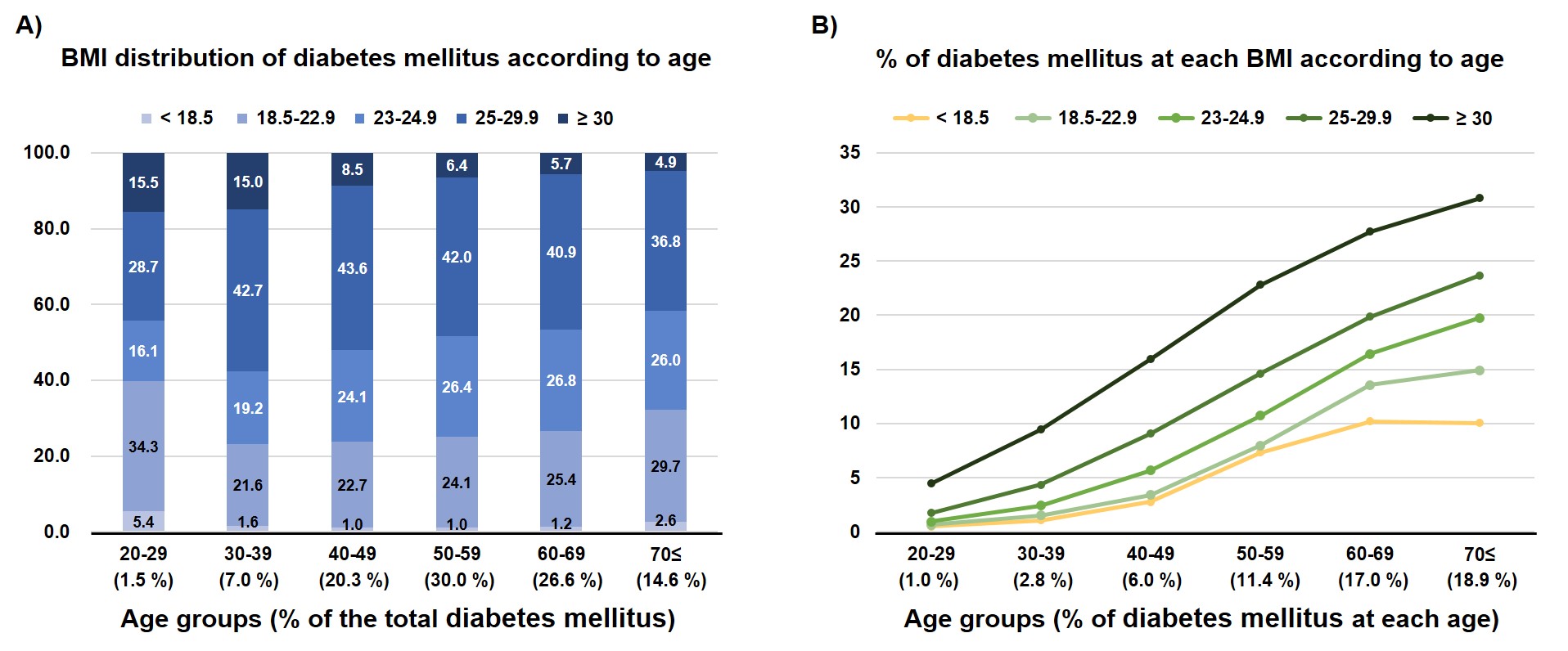


Supplementary Figure 6. A) The BMI distribution of hypertension, B) proportion of hypertension at each BMI according to age

Abbreviation: BMI, body mass index.


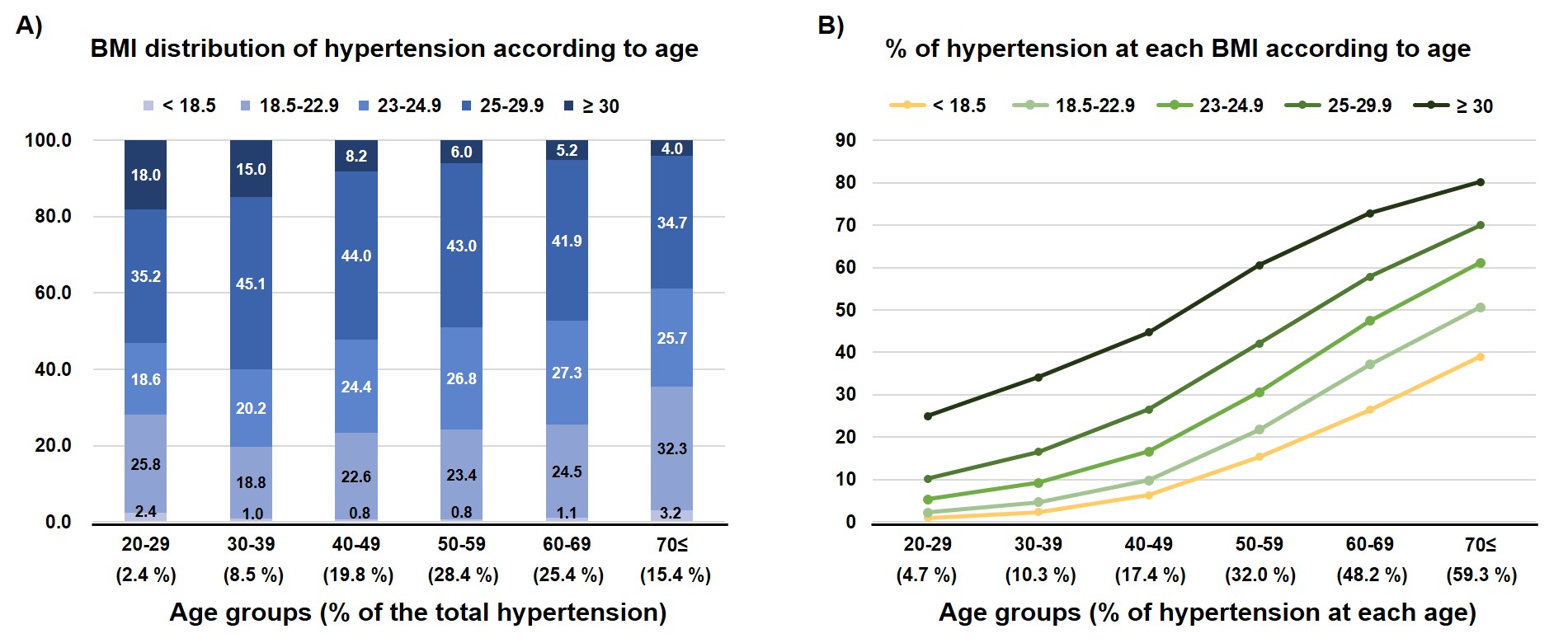


Supplementary Figure 7. A) The BMI distribution of dyslipidemia, B) proportion of dyslipidemia at each BMI according to age

Abbreviation: BMI, body mass index.


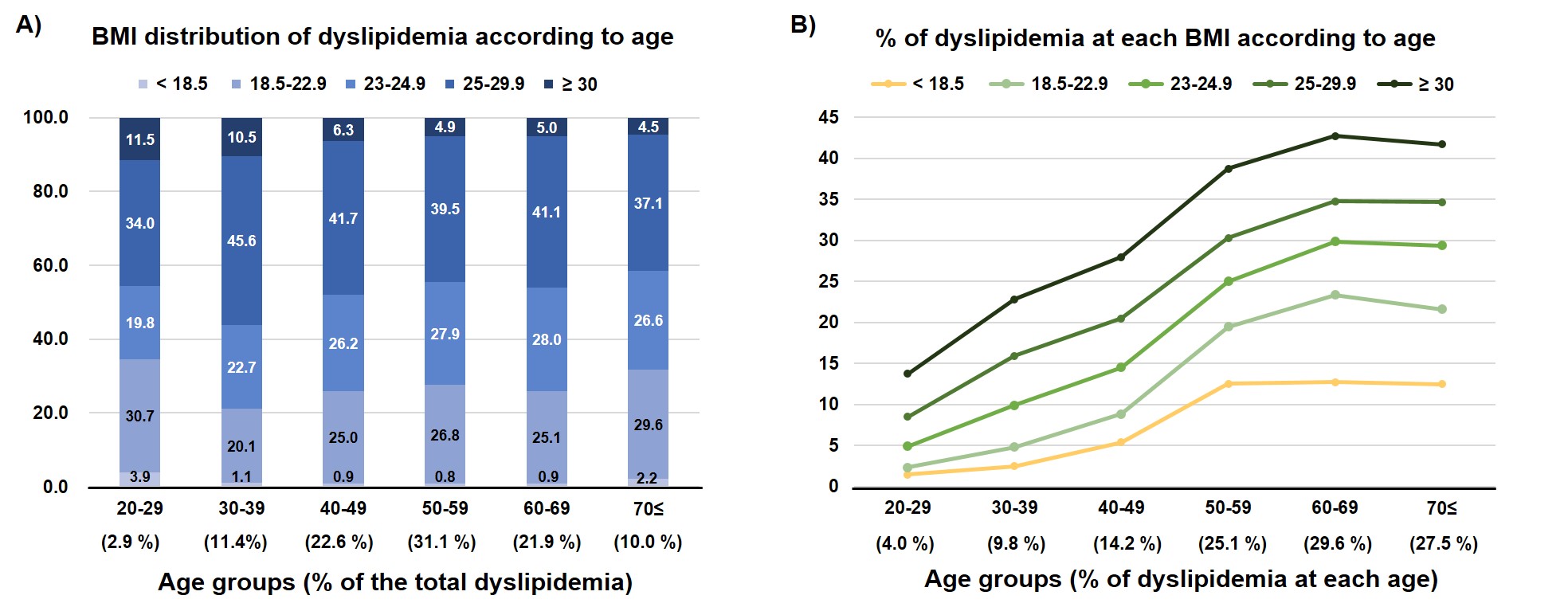


Supplementary Figure 8. A) The BMI distribution of CKD, B) proportion of CKD at each BMI according to age

Abbreviations: BMI, body mass index; CKD, chronic kidney disease.


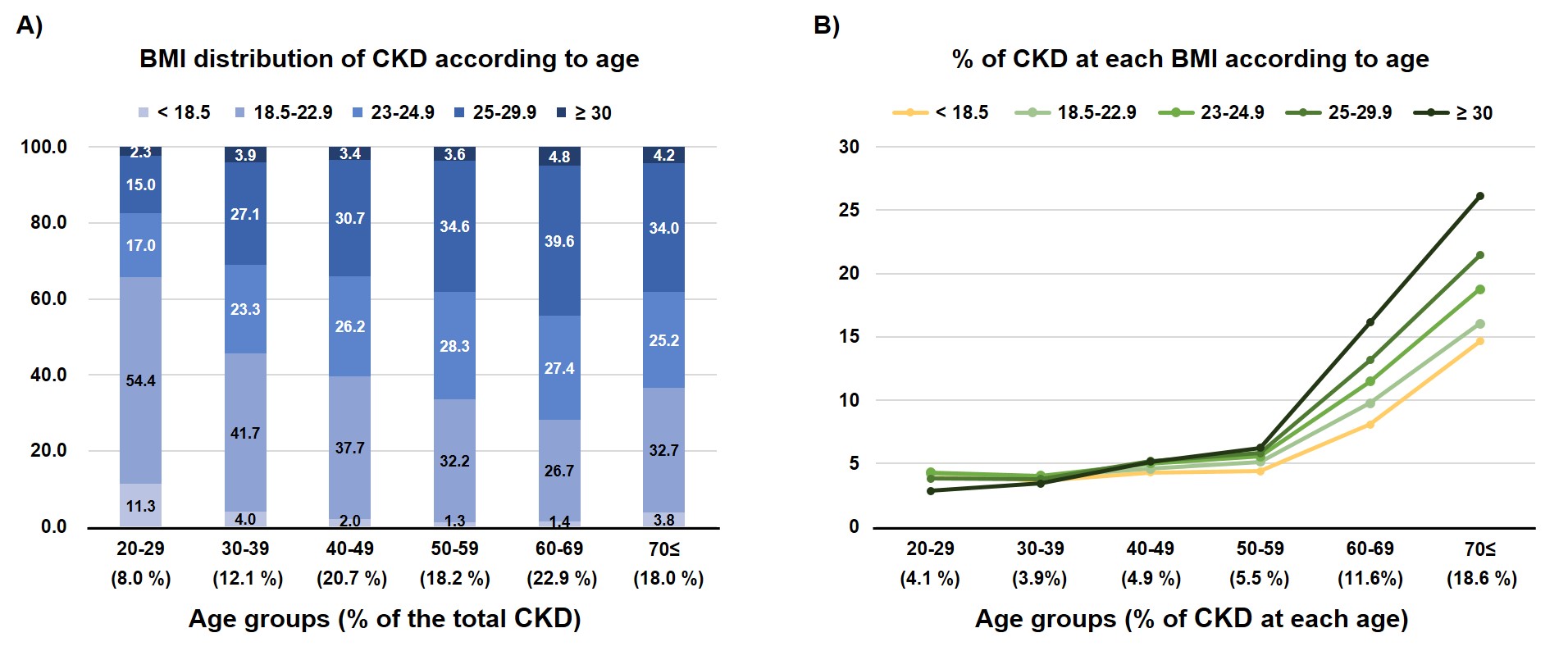


Supplementary Figure 9. A) The BMI distribution of MI, B) proportion of MI at each BMI according to age

Abbreviations: BMI, body mass index; MI, myocardial infarction.


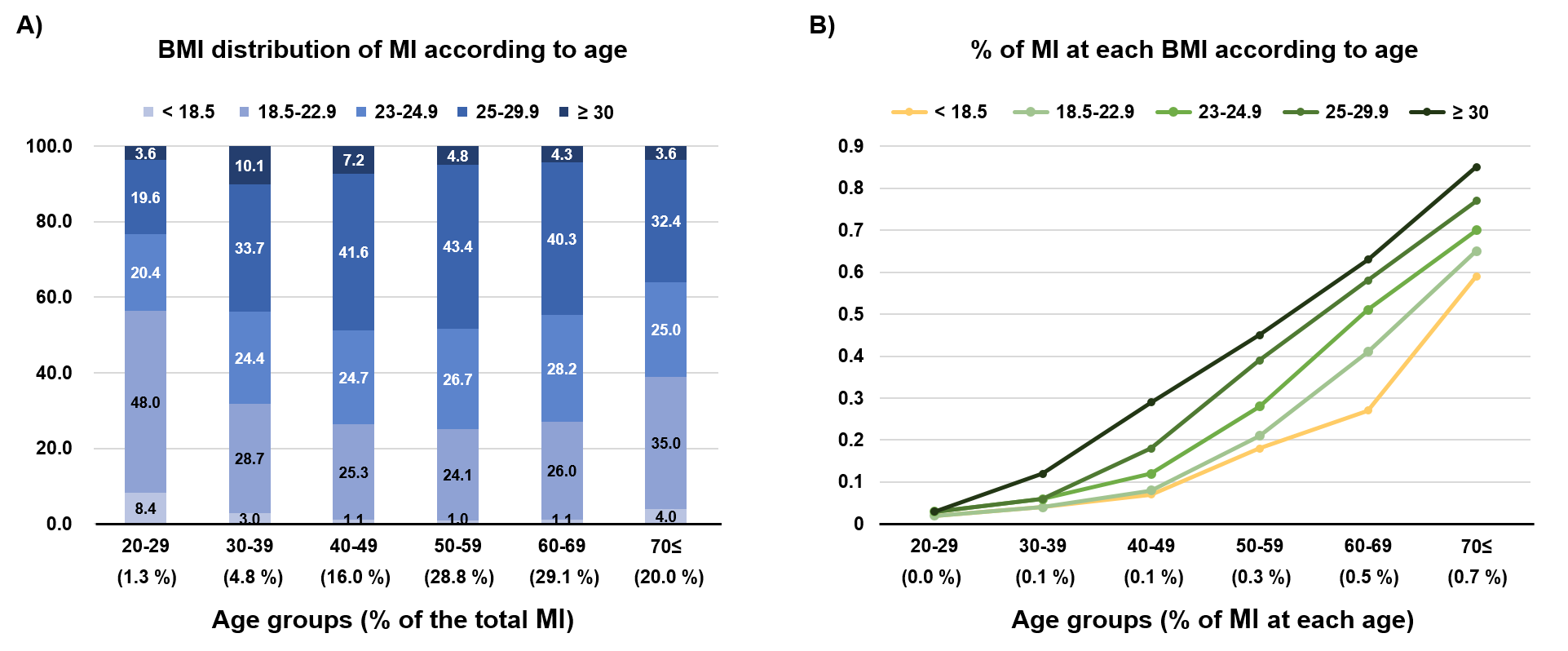


Supplementary Figure 10. A) The BMI distribution of PAD, B) proportion of PAD at each BMI according to age

Abbreviations: BMI, body mass index; PAD, peripheral artery disease.


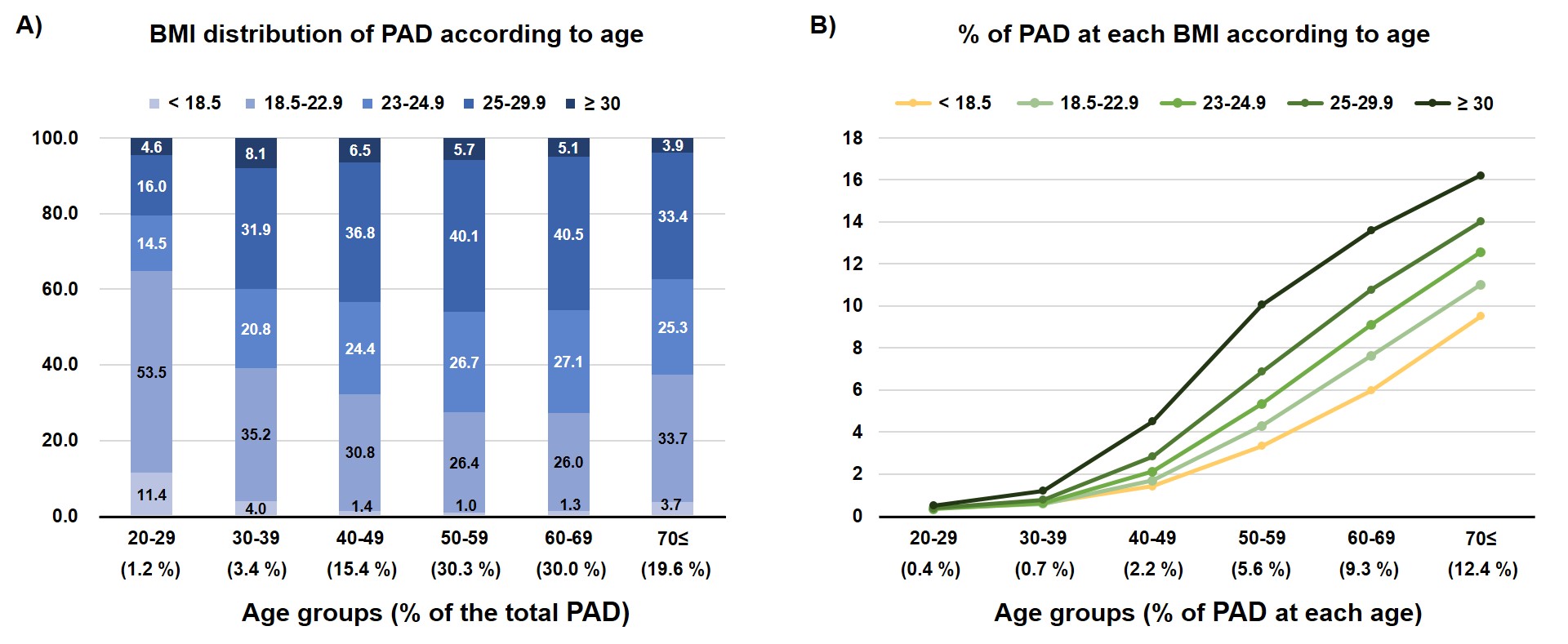


Supplementary Figure 11. A) The BMI distribution of COPD, B) proportion of COPD at each BMI according to age

Abbreviations: BMI, body mass index; COPD, chronic obstructive pulmonary disease.


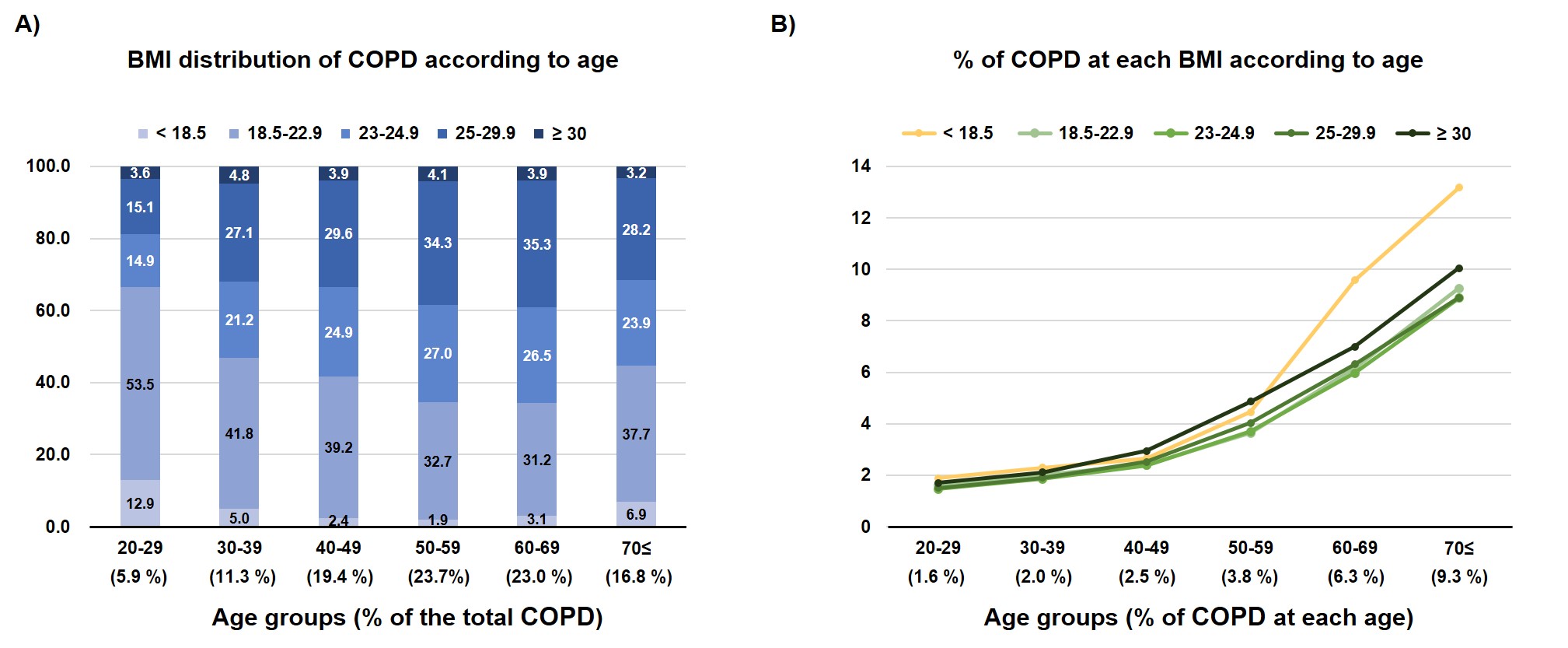


Supplementary Figure 12. A) The BMI distribution of cancer, B) proportion of cancer at each BMI according to age

Abbreviation: BMI, body mass index.


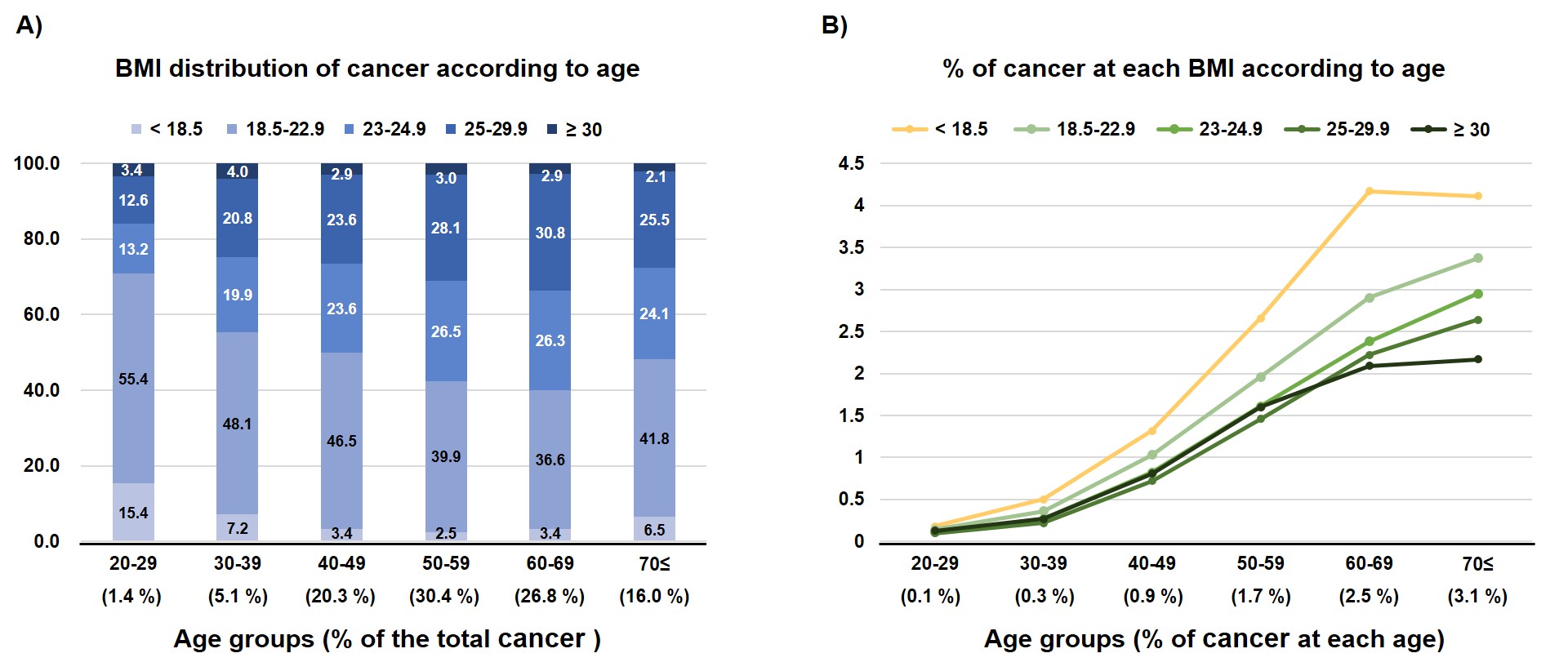


Supplementary Figure 13. A) The BMI distribution of LC, B) proportion of LC at each BMI according to age

Abbreviations: BMI, body mass index; LC, liver cirrhosis.


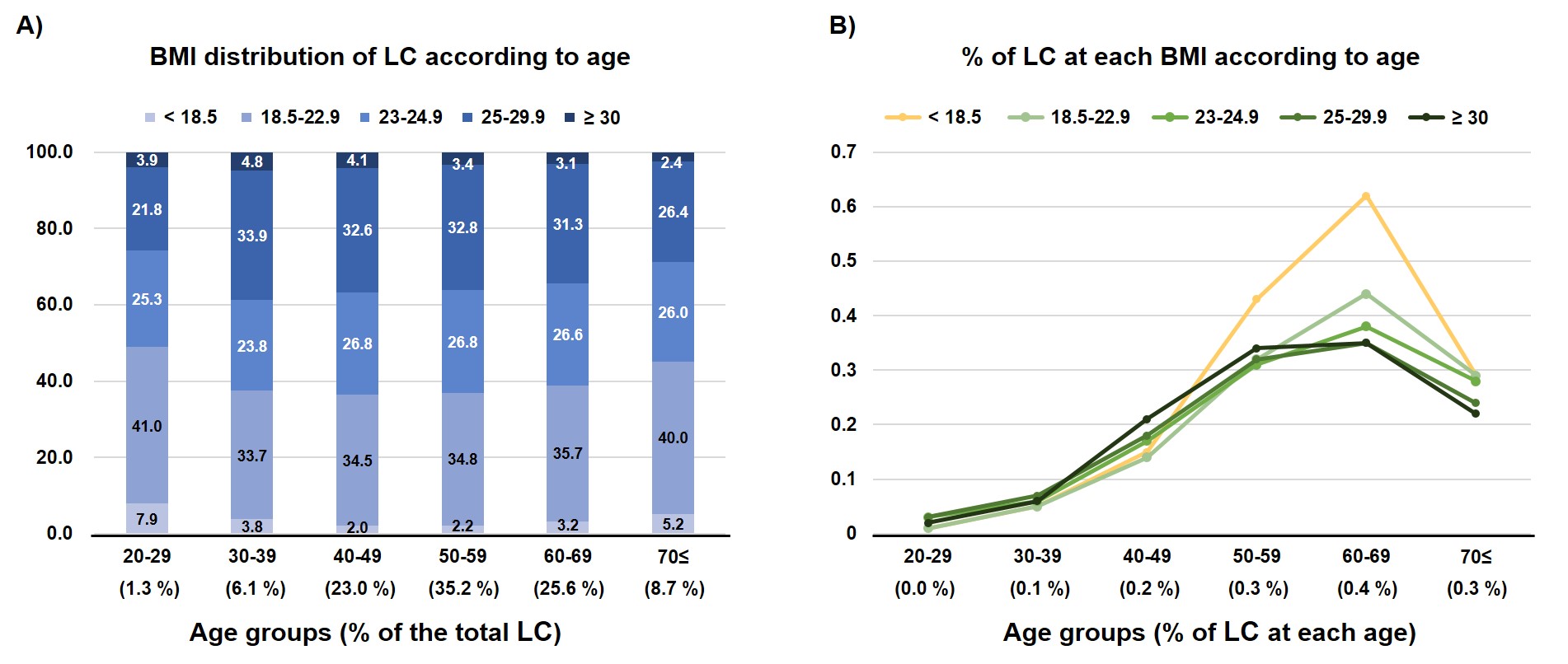


Supplementary Figure 14. A) The BMI distribution of low income, B) proportion of low income at each BMI according to age

Abbreviation: BMI, body mass index.


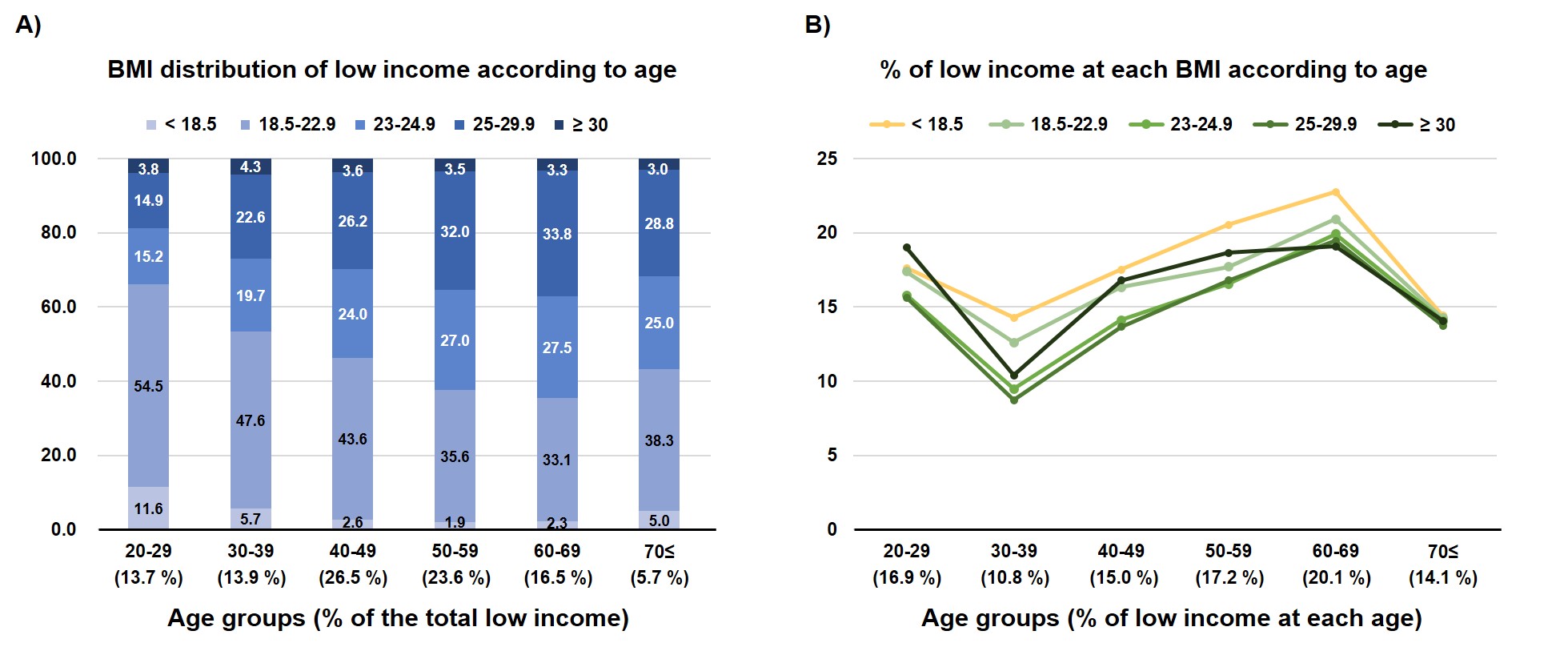


Supplementary Figure 15. The association between body mass index and the risk of AF according to age differentiated by sex.


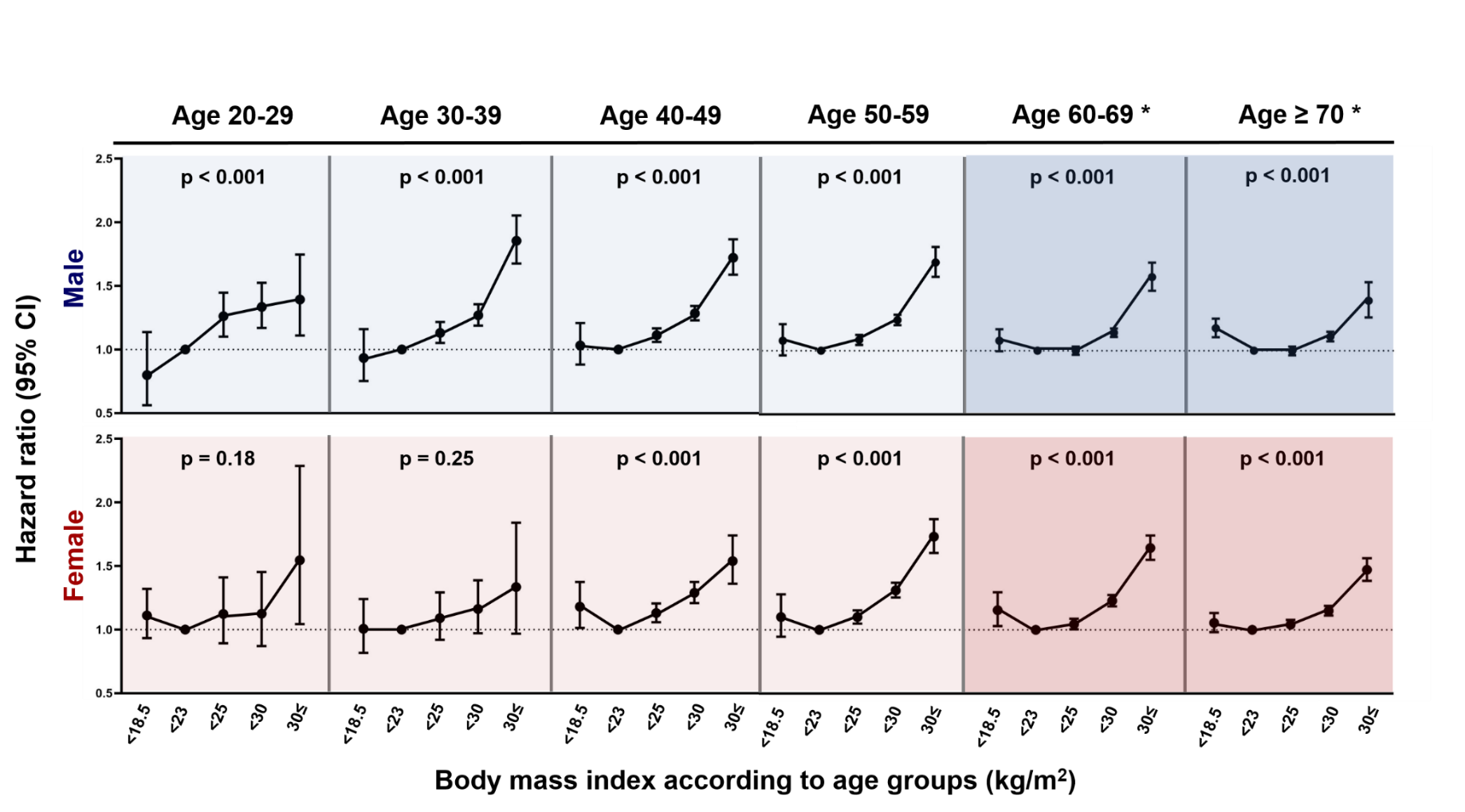


* Age group with P-for-interaction < 0.05

Abbreviation: CI, confidence interval.

P-values indicate the statistical significance of the association between body mass index and the risk of atrial fibrillation in each age group by computing body mass index as continuous variables and adjusting covariates noted in Model 3.

Supplementary Figure 16. The association between waist circumference and the risk of atrial fibrillation according to age differentiated by sex.


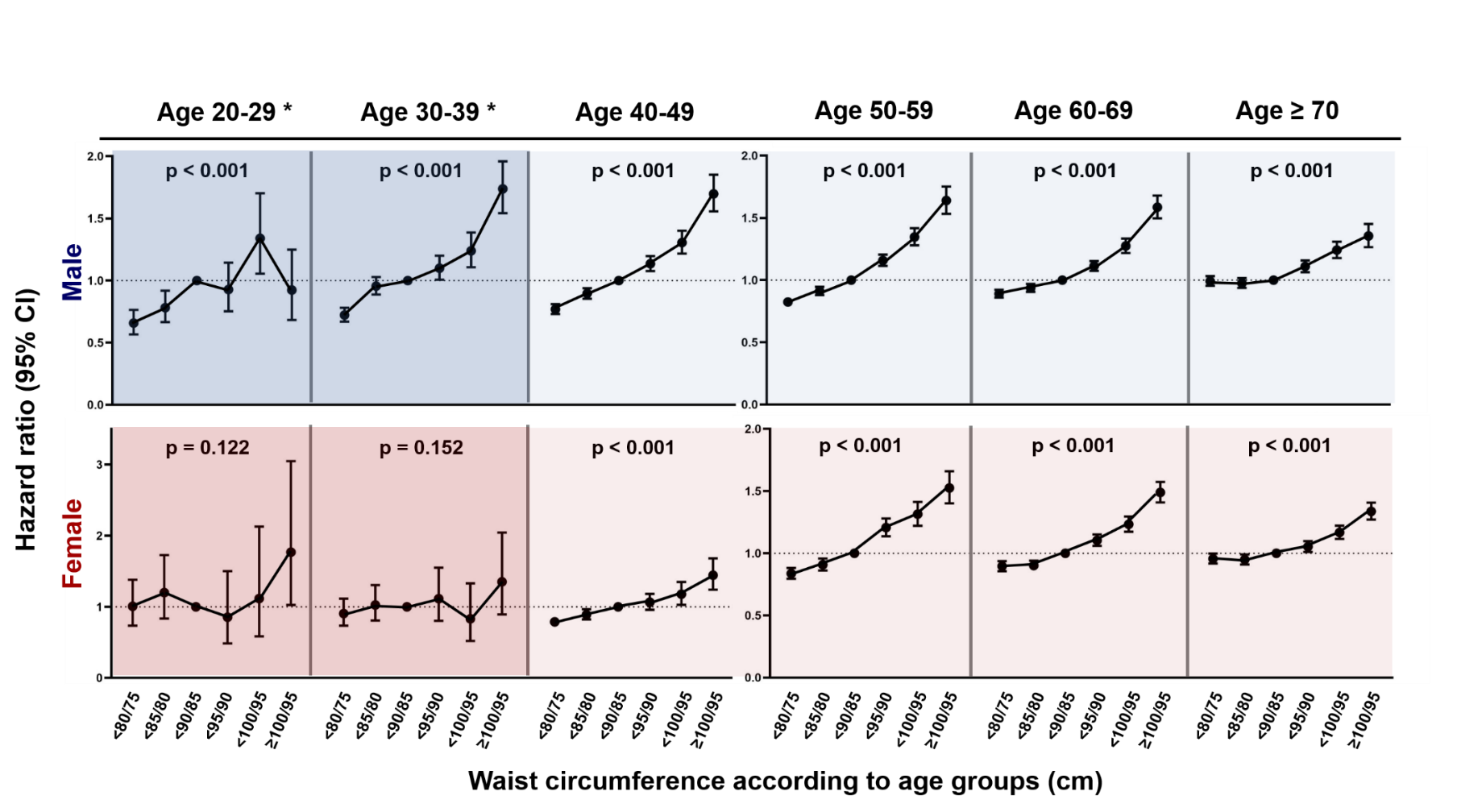


* Age group with P-for-interaction < 0.05

Abbreviation: CI, confidence interval.

P-values indicate the statistical significance of the association between waist circumference and the risk of atrial fibrillation in each age group by computing waist circumference as continuous variables and adjusting covariates noted in Model 3.

Supplementary Figure 17. The association between body mass index and the risk of ischaemic stroke according to age differentiated by sex.


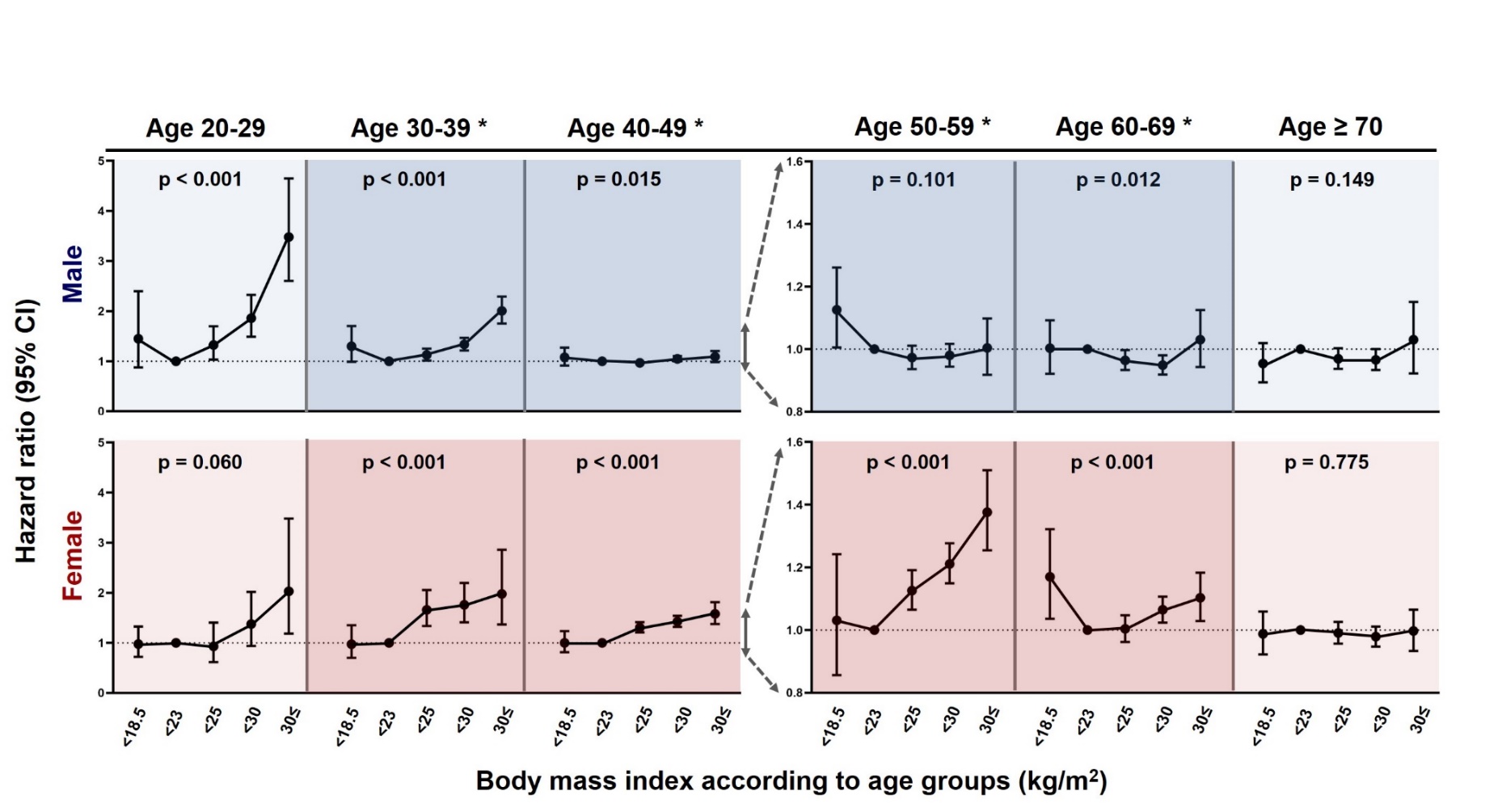


* Age group with P-for-interaction < 0.05

Abbreviation: CI, confidence interval.

P-values indicate the statistical significance of the association between body mass index and the risk of ischemic stroke in each age group by computing body mass index as continuous variables and adjusting covariates noted in Model 3.

Supplementary Figure 18. The association between waist circumference and the risk of ischaemic stroke according to age differentiated by sex.


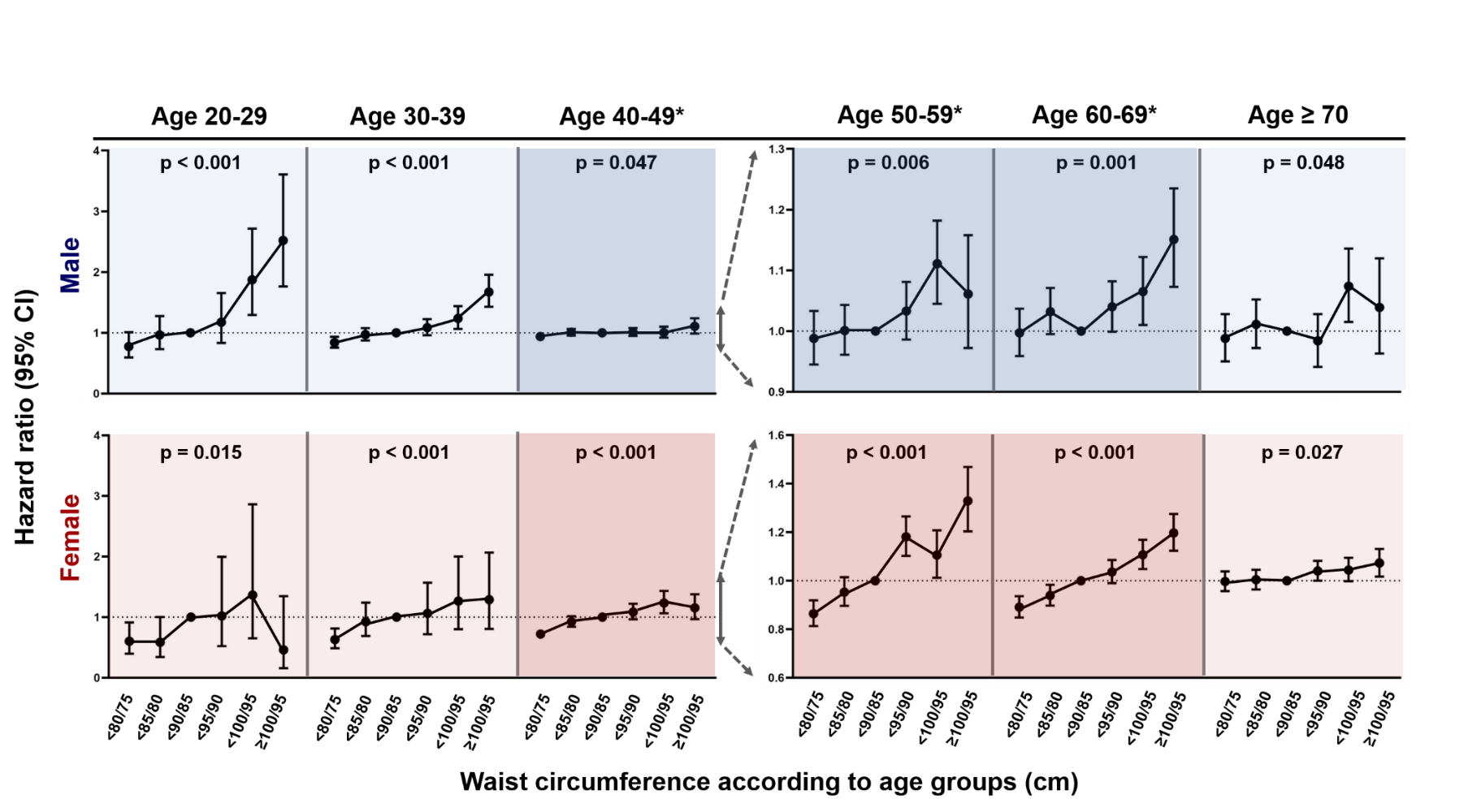


* Age group with P-for-interaction < 0.05

Abbreviation: CI, confidence interval.

P-values indicate the statistical significance of the association between waist circumference and the risk of ischemic stroke in each age group by computing waist circumference as continuous variables and adjusting covariates noted in Model 3.

Supplementary Figure 19. The association between obesity measurements (BMI, WC) and the risk of atrial fibrillation excluding individuals diagnosed with atrial fibrillation within the first two years of follow-up


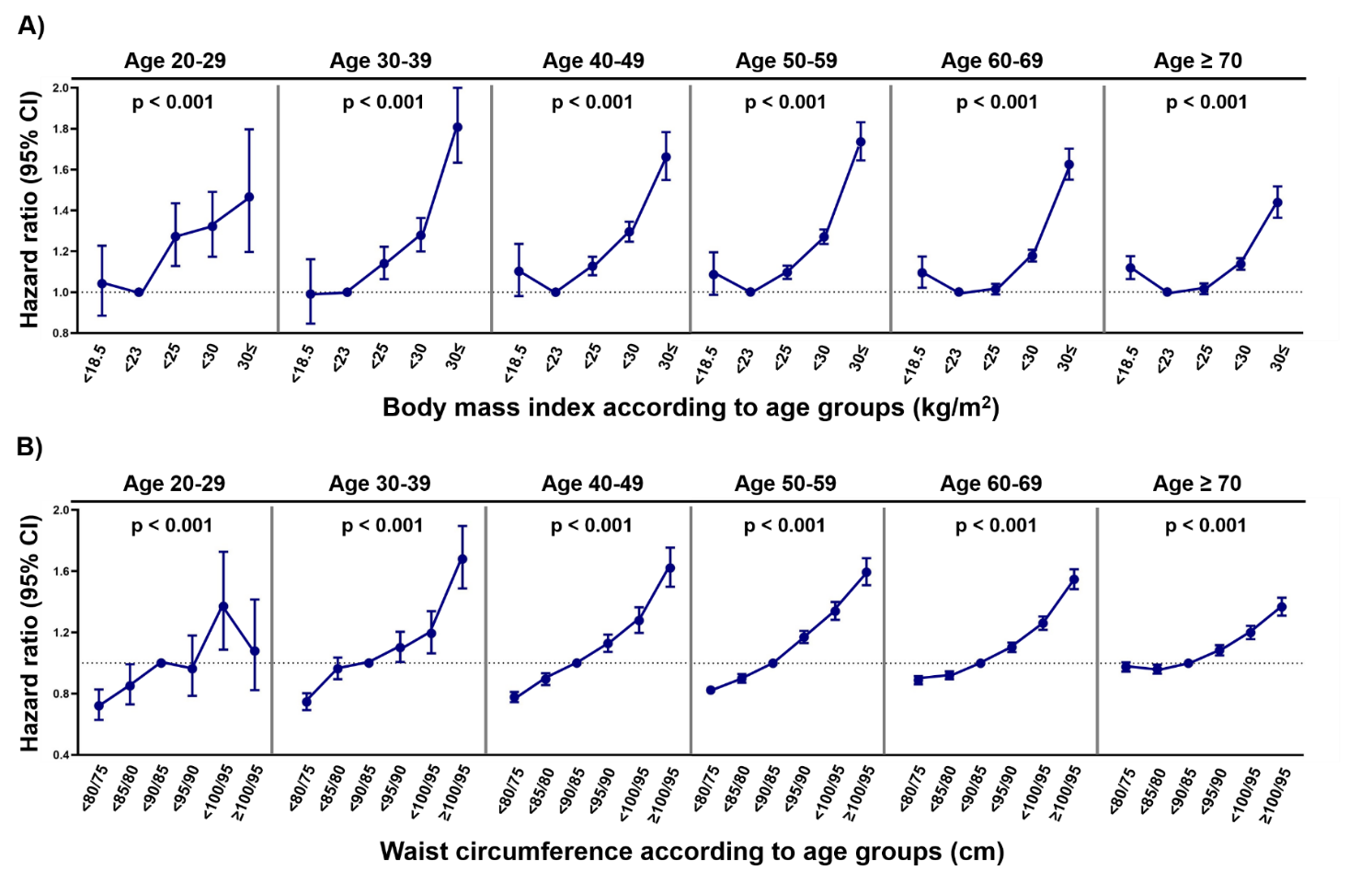


Abbreviation: CI, confidence interval

P-values indicate the statistical significance of the association between obesity parameters (body mass index, waist circumference) and the risk of atrial fibrillation in each age group by computing obesity parameters as continuous variables and adjusting covariates noted in Model 3.

Supplementary Figure 20. The association between obesity measurements (BMI, WC) and the risk of ischaemic stroke excluding individuals diagnosed with ischaemic stroke within the first two years of follow-up

**
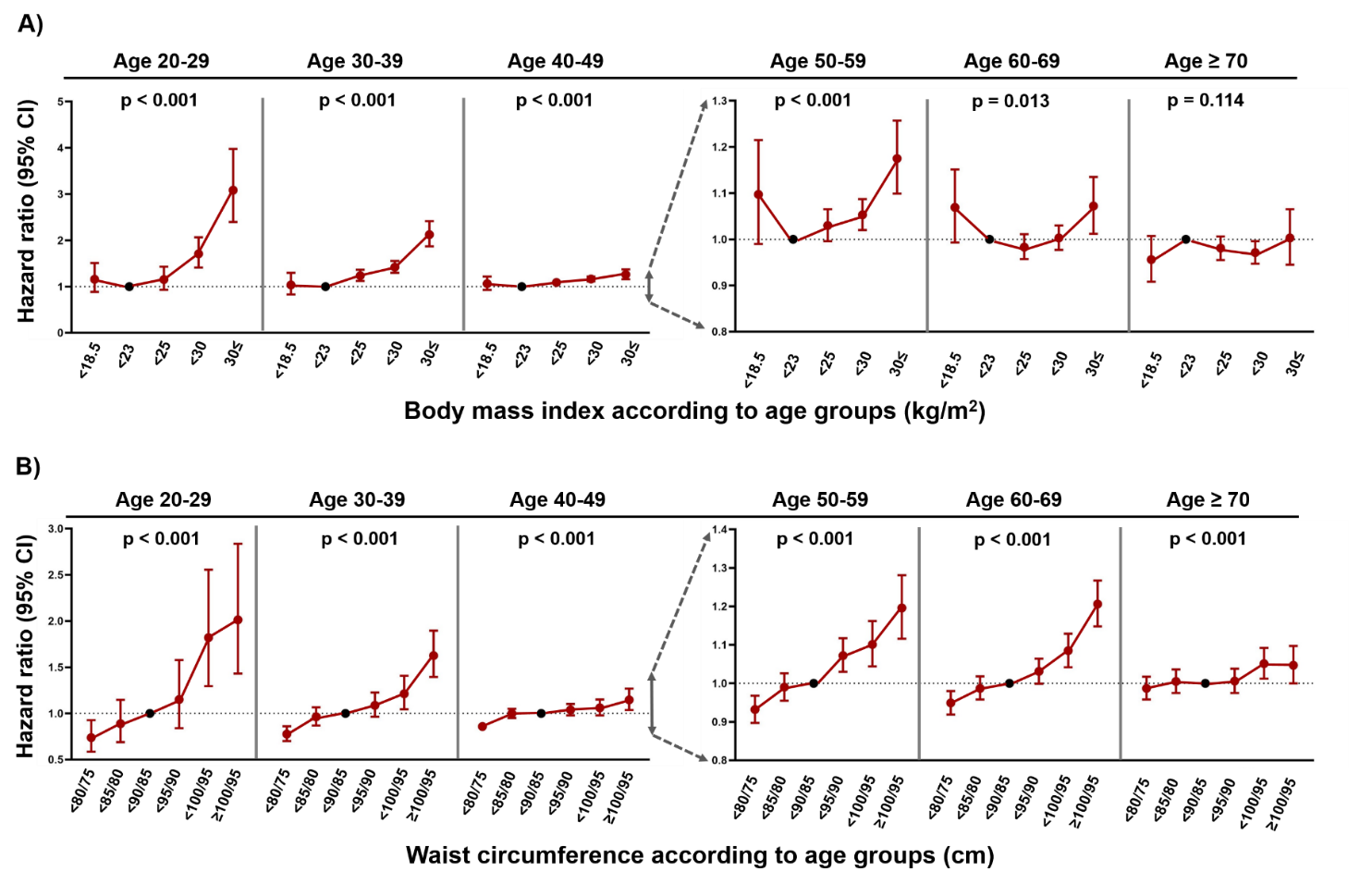
**

Abbreviation: CI, confidence interval

P-values indicate the statistical significance of the association between obesity parameters (body mass index, waist circumference) and the risk of ischemic stroke in each age group by computing obesity parameters as continuous variables and adjusting covariates noted in Model 3.
